# Generalised Entropy Accumulation

**DOI:** 10.1007/s00220-024-05121-4

**Published:** 2024-10-12

**Authors:** Tony Metger, Omar Fawzi, David Sutter, Renato Renner

**Affiliations:** 1 Institute for Theoretical Physics, ETH Zurich, 8093 Zurich, Switzerland; 2 Univ Lyon, Inria, ENS Lyon, UCBL, LIP, 69342 Lyon, France; 3 IBM Quantum, IBM Research Europe, Zurich, Switzerland

## Abstract

Consider a sequential process in which each step outputs a system $A_i$ and updates a side information register *E*. We prove that if this process satisfies a natural “non-signalling” condition between past outputs and future side information, the min-entropy of the outputs $A_1, \dots , A_n$ conditioned on the side information *E* at the end of the process can be bounded from below by a sum of von Neumann entropies associated with the individual steps. This is a generalisation of the entropy accumulation theorem (EAT) (Dupuis et al. in Commun Math Phys 379: 867–913, 2020), which deals with a more restrictive model of side information: there, past side information cannot be updated in subsequent rounds, and newly generated side information has to satisfy a Markov condition. Due to its more general model of side-information, our generalised EAT can be applied more easily and to a broader range of cryptographic protocols. As examples, we give the first multi-round security proof for blind randomness expansion and a simplified analysis of the E91 QKD protocol. The proof of our generalised EAT relies on a new variant of Uhlmann’s theorem and new chain rules for the Rényi divergence and entropy, which might be of independent interest.

## Introduction

Suppose that Alice and Eve share a quantum state $\rho _{A^n E}$. From her systems $A^n {:}{=}A_1 \dots A_n$, Alice would like to extract bits that look uniformly random to Eve, except with some small failure probability $\varepsilon$ [1]. The number of such random bits that Alice can extract is given by the smooth min-entropy $H_\text {min}^\varepsilon (A^n|E)_\rho$ [2]. This quantity plays a central role in quantum cryptography: for example, the main task in security proofs of quantum key distribution (QKD) protocols is usually finding a lower bound for the smooth min-entropy.

Unfortunately, for many cryptographic protocols deriving such a bound is challenging. Intuitively, the reason is the following: the state $\rho _{A^n E}$ is usually created as the output of a multi-round protocol, where each round produces one of Alice’s systems $A_i$ and allows Eve to execute some attack to gain information about $A_1, \dots , A_i$. These attacks can depend on each other, i.e., Eve may use what she learnt in round $i - 1$ to plan her attack in round *i*. This non-i.i.d. nature of the attacks makes it hard to find a lower bound on $H_\text {min}^\varepsilon (A^n|E)_\rho$ that holds for any possible attack that Eve can execute. In contrast, it is typically much easier to compute a conditional von Neumann entropy associated with a single-round of the protocol, where the non-i.i.d. nature of Eve’s attack plays no role. Therefore, it is desirable to relate the smooth min-entropy of the output of the multi-round protocol to the von Neumann entropies associated with the individual rounds.

From an information-theoretic point of view, this question can be phrased as follows: can the smooth min-entropy $H_\text {min}^\varepsilon (A^n | E)_\rho$ be bounded from below in terms of von Neumann entropies $H(A_i | E_i)_{\rho ^i_{A_i E_i}}$ for some (yet to be determined) systems $E_i$ and states $\rho ^i_{A_i E_i}$ related to $\rho$? While for general states $\rho _{A^n E}$ no useful lower bound can be found, previous works have established such bounds under additional assumptions on the state $\rho _{A^n E}$.

The first bound of this form was proven via the *asymptotic equipartition property* (AEP) [3]. It assumes that the system *E* is *n*-partite (i.e., we replace *E* by $E^n = E_1 \dots E_n$) and that the state $\rho _{A^n E^n} = \rho _{A_1 E_1} \otimes \dots \otimes \rho _{A_n E_n}$ is a product of identical states. Then, the AEP shows that^1^

$$\begin{aligned} H_{\min }^{\varepsilon }(A^n | E^n)_\rho \ge \sum _{i=1}^n H(A_i | E_i)_\rho - O(\sqrt{n}) \ . \end{aligned}$$

For applications in cryptography, the assumption that $\rho$ is an i.i.d. product state is usually too strong: it corresponds to the (unrealistic) assumption that Eve executes the same independent attack in each round, a so-called *collective attack*.

The *entropy accumulation theorem* (EAT) [1] is a generalisation of the AEP which requires far weaker assumptions on the state $\rho _{A^nE}$. Specifically, the EAT considers states that result from a sequential process that starts with a state $\rho ^0_{R_0 E'}$ and in every step outputs a system $A_i$ and a piece of side information $I_i$. The system $E'$ is not acted upon during the process. The full side information at the end of this process is $E = I_1 \dots I_n E'$. We can represent such a process by the following diagram, where $\mathcal {M}_i$ are quantum channels. 
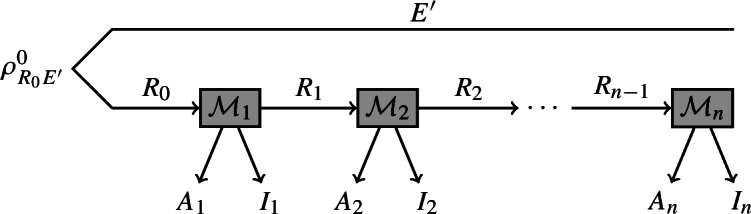
 The EAT requires an additional condition on the side information: the new side information $I_i$ generated in round *i* must be independent from the past outputs $A^{i-1}$ conditioned on the existing side information $I^{i-1} E'$. Mathematically, this is captured by the condition that the systems $A^{i-1} \leftrightarrow I^{i-1} E' \leftrightarrow I_i$ form a Markov chain for any initial state $\rho ^0_{R_0 E'}$. With this Markov condition, the EAT states that^2^

1.1 $$\begin{aligned} H_{\min }^{\varepsilon }(A^n | I^n E')_{\mathcal {M}_n \circ \dots \circ \mathcal {M}_1(\rho ^0_{R_0 E' })} \ge \sum _{i=1}^n \inf _{\omega } H(A_i | I_i {{\tilde{E}}})_{\mathcal {M}_i(\omega )} - O(\sqrt{n}) \ , \end{aligned}$$

where ${{\tilde{E}}}$ is a purifying system isomorphic to $R_{i-1}$ and the infimum is taken over all states $\omega$ on systems $R_{i-1}{{\tilde{E}}}$.^3^

Let us discuss the model of side information used by the EAT in more detail. The EAT considers side information consisting of two parts: the initial side information $E'$ (which is not acted upon during the process) and the outputs $I^n = I_1 \dots I_n$. This splitting of side information into a “static” part $E'$ and a part $I^n$ which is generated in each step of the process is particularly suited to device-independent cryptography: there, Eve prepares a device in an initial state $\rho ^0_{R_0 E'}$, where $R_0$ is the device’s internal memory and $E'$ is Eve’s initial side information from preparing the device. Then, Alice (and Bob, though we only consider Alice’s system here) executes a multi-round protocol with this device, where each round leaks some additional piece of information $I_i$ to Eve, so that Eve’s side information at the end of the protocol is $I^n E'$. Indeed, the EAT has been used to establish tight security proofs in the device-independent setting, see e.g., [4, 5].

The Markov condition in the EAT captures the following intuition: if we want to find a bound on $H_{\min }^{\varepsilon }(A^n | I^n E')$ in terms of single-round quantities, it is required that side information about $A_i$ is itself output in step *i*, as otherwise we cannot hope to estimate the contribution to the total entropy from step *i*. To illustrate what could happen without such a condition, consider a case where $A_i$ is classical and no side information is output in the first $n-1$ rounds, but the side information $I_n$ in the last round contains a copy of the systems $A^n$ (which can be passed along during the process in the systems $R_i$). Then, clearly $H_{\min }^{\varepsilon }(A^n | I^n E') = 0$, but for the first $n-1$ rounds, each single-round entropy bound that only considers the systems $A_i$ and $I_i$ can be positive.

*Main result* In this work, we further relax the assumptions on how the final state $\rho _{A^n E}$ is generated. Specifically, we consider sequential processes as in the EAT, but with a fully general model of side information, i.e., the side information can be updated in each step in the process. Diagrammatically, such a process can be represented as follows:
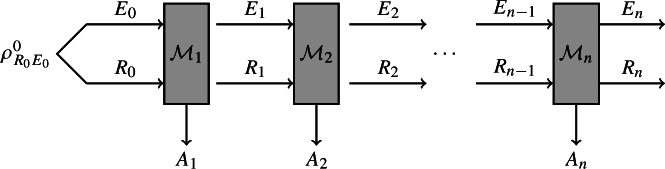


Our generalised EAT then states the following.

### Theorem 1.1

Consider quantum channels $\mathcal {M}_i: R_{i-1} E_{i-1} \rightarrow A_i R_i E_i$ that satisfy the following “non-signalling” condition (discussed in detail below): for each $\mathcal {M}_i$, there must exist a quantum channel $\mathcal {R}_i: E_{i-1} \rightarrow E_i$ such that

1.2 $$\begin{aligned} \text {Tr}_{A_i R_i} \circ \mathcal {M}_i = \mathcal {R}_i \circ \text {Tr}_{R_{i-1}} \,. \end{aligned}$$

Then, the min-entropy of the outputs $A^n$ conditioned on the final side information $E_n$ can be bounded as

1.3 $$\begin{aligned} H_\text {min}^\varepsilon (A^n | E_n )_{\mathcal {M}_n \circ \dots \circ \mathcal {M}_1(\rho ^0_{R_0 E_0 })} \ge \sum _{i = 1}^n \inf _{\omega } H(A_i|E_i {{\tilde{E}}}_{i-1} )_{\mathcal {M}_i(\omega )} - O(\sqrt{n}) \, , \end{aligned}$$

where ${{\tilde{E}}}_{i-1} \equiv R_{i-1}E_{i-1}$ is a purifying system for the input to $\mathcal {M}_i$ and the infimum is taken over all states $\omega$ on systems $R_{i-1}E_{i-1}{{\tilde{E}}}_{i-1}$.^4^

We give a formal statement and proof in Sect. 4 and also show that, similarly to the EAT, statistics collected during the process can be used to restrict the minimization over $\omega$ (see Theorem 4.3 for the formal statement). By a simple duality argument, Eq. (1.3) also implies an upper bound on the smooth max-entropy $H_{\text {max}}$, which we explain in Appendix A. This generalises a similar result from [1], although in [1] one could not make use of duality due to the Markov condition and instead had to prove the statement about $H_{\text {max}}$ separately, again highlighting that our generalised EAT is easier to work with.

The intuition behind the non-signalling condition in our generalised EAT is similar to the Markov condition in the original EAT: by the same reasoning as for the Markov condition, since the lower bound is made up of terms of the form $H(A_i|E_i {{\tilde{E}}}_{i-1} )_{\mathcal {M}_i(\omega )}$, it is required that side information about $A_i$ that is present in the final system $E_n$ is already present in $E_i$. This means that side information about $A_i$ should not be passed on via the *R*-systems and later be included in the *E*-systems. The non-signalling condition captures this requirement: it demands that if one only considers the marginal of the new side information $E_i$ (without the new output $A_i$), it must be possible to generate this state from the past side information $E_{i-1}$ alone, without access to the system $R_{i-1}$. This means that any side information that $E_i$ contains about the past outputs $A_1 \dots A_{i-1}$ must have essentially already been present in $E_{i-1}$ and could not have been stored in $R_{i-1}$.

The name “non-signalling condition” is due to the fact that Eq. (1.2) is a natural generalisation of the standard non-signalling conditions in non-local games: if we view the systems $R_{i-1}$ and $R_i A_i$ as the inputs and outputs on “Alice’s side” of $\mathcal {M}_i$, and $E_{i-1}$ and $E_i$ as the inputs and outputs on “Eve’s side”, then Eq. (1.2) states that the marginal of the output on Eve’s side cannot depend on the input on Alice’s side. This is exactly the non-signalling condition in non-local games, except that here the inputs and outputs are allowed to be fully quantum.

To understand the relation between the Markov and non-signalling conditions, it is instructive to consider the setting of the original EAT as a special case of our generalised EAT. In the original EAT, the full side information available after step *i* is $E' I^{i}$, and past side information is not updated during the process. For our generalised EAT, we therefore set $E_i = E' I^i$ and consider maps $\mathcal {M}_i = \mathcal {M}'_i \otimes \text {id}_{E_{i-1}}$, where $\mathcal {M}'_i: R_{i-1} \rightarrow A_i I_i R_i$ is the map used in the original EAT. We need to check that with this choice of systems and maps, the Markov condition of the original EAT implies the non-signalling condition of our generalised EAT. The Markov condition requires that for any state input $\omega ^{i-1}_{A^{i-1}I^{i-1}R_{i-1}E'}$, the output state $\omega ^{i}_{A^i I^i R_i E'} = \mathcal {M}_{i}(\omega ^{i-1})$ satisfies $A^{i-1} \leftrightarrow I^{i-1} E' \leftrightarrow I_i$.^5^ It is then a standard result on quantum Markov chains [6] that there must exist a quantum channel $\mathcal {R}_i: I^{i-1} E' \rightarrow I^{i} E'$ such that $\omega ^i_{I^i E'} = \mathcal {R}_i(\omega ^{i-1}_{I^{i-1} E'})$. Remembering that we defined $E_i = E' I^i$ (so that $\mathcal {R}_i: E_{i-1} \rightarrow E_i$) and adding the systems $A^{i-1}$ (on which both $\mathcal {M}_i$ and $\mathcal {R}_i$ act as identity), we find that $\mathcal {M}_i$ satisfies the non-signalling condition:

$$\begin{aligned} \text {Tr}_{A_i R_i} \circ \mathcal {M}_i(\omega ^{i-1}_{A^{i-1} R_{i-1} E_{i-1}}) = \omega ^i_{A^{i-1} E_i} = \mathcal {R}_i(\omega ^{i-1}_{A^{i-1} E_{i-1}}) = \mathcal {R}_i \circ \text {Tr}_{R_{i-1}} (\omega ^{i-1}_{A^{i-1} R_{i-1} E_{i-1}}) \,. \end{aligned}$$

Then, noting that all conditioning systems on which $\mathcal {M}_i$ acts as the identity map can collectively be replaced by a single purifying system isomorphic to the input, we see that we recover the original EAT (Eq. (1.1)) from our generalised EAT (Eq. (1.3)).

We emphasise that while the original EAT with the Markov condition can be recovered as a special case, our model of side information and the non-signalling condition are much more general than the original EAT; arguably, for a sequential process they are the most natural and general way of expressing the notion that future side information should not contain new information about past outputs, which appears to be necessary for an EAT-like result. To demonstrate the greater generality of our result, in Sect. 5 we use it to give the first multi-round proof for blind randomness expansion, a task to which the original EAT could not be applied, and a more direct proof of the E91 QKD protocol than was possible with the original EAT. Our generalised EAT can also be used to prove security of a much larger class of QKD protocols than the original EAT. Interestingly, for (device-dependent) QKD protocols, no “hidden system” *R* is needed and therefore the non-signalling condition is trivially satisfied, i.e., the advantage of our generalised EAT for QKD security proofs stems entirely from the more general model of side information, not from replacing the Markov condition by the non-signalling condition; see Sect. 5.2 for an informal comparison of how the original and generalised EAT can be applied to QKD, and [7] for a detailed treatment of the application of our generalised EAT to QKD, including protocols to which the original EAT could not be applied.

*Proof sketch.* The generalised EAT involves both the min-entropy, which can be viewed as a “worst-case entropy”, and the von Neumann entropy, which can be viewed as an “average case entropy”. These two entropies are special cases of a more general family of entropies called Rényi entropies, which are denoted by $H_\alpha$ for a parameter $\alpha > 1$ (see Sect. 2.2 for a formal definition).^6^ The min-entropy can be obtained from the Rényi entropy by taking $\alpha \rightarrow \infty$, whereas the von Neumann entropy corresponds to the limit $\alpha \rightarrow 1$. Hence, the Rényi entropies interpolate between the min-entropy and the von Neumann entropy, and they will play a crucial role in our proof.

The key technical ingredient for our generalised EAT is a new chain rule for Rényi entropies (Theorem 3.6 in the main text).

### Lemma 1.2

Let $\alpha \in (1, 2)$, $\rho _{ARE}$ a quantum state, and $\mathcal {M}: RE \rightarrow A'R'E'$ a quantum channel which satisfies the non-signalling condition in Eq. (1.2), i.e. there exists a channel $\mathcal {R}: E \rightarrow E'$ such that $\text {Tr}_{A'R'} \circ \mathcal {M}= \mathcal {R}\circ \text {Tr}_{R}$. Then

1.4 $$\begin{aligned} H_{\alpha }(AA'|E')_{\mathcal {M}(\rho )} \ge H_{\alpha }(A|E)_{\rho } + \inf _{\omega _{RE{{\tilde{E}}}}}H_{\frac{1}{2-\alpha }}(A'|E' \tilde{E})_{\mathcal {M}(\omega )} \end{aligned}$$

for a purifying system ${{\tilde{E}}} \equiv RE$, where the infinimum is over all quantum states $\omega$ on systems $RE{{\tilde{E}}}$.

We first describe how this chain rule implies our generalised EAT, following the same idea as in [1, 8]. For this, recall that our goal is to find a lower bound on $H_\text {min}^\varepsilon (A^n | E_n )_{\mathcal {M}_n \circ \dots \circ \mathcal {M}_1(\rho ^0_{R_0 E_0 })}$ for a sequence of maps satisfying the non-signalling condition $\text {Tr}_{A_i R_i} \circ \mathcal {M}_i = \mathcal {R}_i \circ \text {Tr}_{R_{i-1}}$. As a first step, we use a known relation between the smooth min-entropy and the Rényi entropy [3], which (up to a small penalty term depending on $\varepsilon$ and $\alpha$) reduces the problem to lower-bounding

$$\begin{aligned} H_\alpha (A^n | E_n )_{\mathcal {M}_n \circ \dots \circ \mathcal {M}_1(\rho ^0_{R_0 E_0})} = H_\alpha (A_n A^{n-1} | E_n )_{\mathcal {M}_n \circ \dots \circ \mathcal {M}_1(\rho ^0_{R_0 E_0})} \,. \end{aligned}$$

To this, we can apply Lemma 1.2 by choosing $A = A^{n-1}$, $A' = A_n$, $E = E_{n-1}$, $E' = E_n$, $R = R_{n-1}$, $R' = R_n$, and $\rho = \mathcal {M}_{n-1} \circ \dots \circ \mathcal {M}_1(\rho ^0_{R_0 E_0})$. Then, since the map $\mathcal {M}_n$ satisfies the non-signalling condition, Lemma 1.2 implies that

$$\begin{aligned} H_{\alpha }(A_1^n | E_n )_{\mathcal {M}_n \circ \dots \circ \mathcal {M}_1(\rho _{R_0 E_0 })}&\ge H_{\alpha }(A_1^{n-1} | E_{n-1} )_{\mathcal {M}_{n-1} \circ \dots \circ \mathcal {M}_1(\rho _{R_0 E_0 })} \\&\quad \ + \inf _{\omega \in \text {S}(R_{n-1}E_{n-1}{{\tilde{E}}}_{n-1})} H_{\frac{1}{2-\alpha }}(A_n|E_n {{\tilde{E}}}_{n-1})_{\mathcal {M}_n(\omega )} \,. \end{aligned}$$

We can now repeat this argument for the term $H_{\alpha }(A_1^{n-1} | E_{n-1} )_{\mathcal {M}_{n-1} \circ \dots \circ \mathcal {M}_1(\rho _{R_0 E_0 })}$. After *n* applications of Lemma 1.2, we find that

$$\begin{aligned} H_{\alpha }(A_1^n | E_n )_{\mathcal {M}_n \circ \dots \circ \mathcal {M}_1(\rho _{R_0 E_0 })}&\ge \sum _{i = 1}^n \inf _{\omega \in \text {S}(R_{i-1}E_{i-1}{\tilde{E}}_{i-1})} H_{\frac{1}{2-\alpha }}(A_i|E_i {\tilde{E}}_{i-1})_{\mathcal {M}_i(\omega )} \,. \end{aligned}$$

To conclude, we use a continuity bound from [8] to relate $H_{\frac{1}{2-\alpha }}(A_i|E_i \tilde{E}_{i-1})_{\mathcal {M}_i(\omega )}$ to $H(A_i|E_i \tilde{E}_{i-1})_{\mathcal {M}_i(\omega )}$. It can be shown that for a suitable choice of $\alpha$, the penalty terms we incur by switching from the min-entropy to the Rényi entropy and then to the von Neumann entropy scale as $O(\sqrt{n})$. Therefore, we obtain Eq. (1.3). We also provide a version that allows for “testing” (which is crucial for application in quantum cryptography and explained in detail in Sect. 4.2) and features explicit second-order terms similar to those in [8].

We now turn our attention to the proof of Lemma 1.2. For this, we need to introduce the (sandwiched) Rényi divergence of order $\alpha$ between two (possibly unnormalised) quantum states $\rho$ and $\sigma$, denoted by $D_\alpha \! \left( \rho \, \big \Vert \, \sigma \right)$. We refer to Sect. 2.2 for a formal definition; for this overview, it suffices to know that $D_\alpha \! \left( \rho \, \big \Vert \, \sigma \right)$ is a measure of how different $\rho$ is from $\sigma$, and that the conditional Rényi entropy is related to the Rényi divergence by

$$\begin{aligned} H_{\alpha }(A|B)_{\rho } = - D_\alpha \! \left( \rho _{AB} \, \big \Vert \, {\mathbb {1}}_A \otimes \rho _B \right) \,. \end{aligned}$$

Our starting point for proving Lemma 1.2 is the following chain rule for the Rényi divergence from [9]:

1.5 $$\begin{aligned} D_\alpha \! \left( \mathcal {M}(\rho ) \, \big \Vert \, \mathcal {F}(\sigma ) \right) \le D_\alpha \! \left( \rho _{ARE} \, \big \Vert \, \sigma _{ARE} \right) + \lim _{n \rightarrow \infty } \frac{1}{n} \sup _{\omega _{R^n E^n {{\tilde{E}}}^n}}D_\alpha \! \left( \mathcal {M}^{\otimes n}(\omega ) \, \big \Vert \, \mathcal {F}^{\otimes n}(\omega ) \right) \,, \end{aligned}$$

where $\mathcal {M}$ and $\mathcal {F}$ are (not necessarily trace preserving) quantum channels from *RE* to $A'R'E'$, and $\rho$ and $\sigma$ are any quantum states on *ARE*. The optimization is over all quantum states $\omega$ on *n* copies of the systems $RE{{\tilde{E}}}$ (with ${{\tilde{E}}} \equiv RE$ as before).

Making a suitable choice of $\mathcal {F}$ (which depends on $\mathcal {M}$) and $\sigma$ (which depends on $\rho$), one can turn Eq. (1.5) into the following chain rule for the conditional Rényi entropy:

1.6 $$\begin{aligned} H_{\alpha }(AA'|E')_{\mathcal {M}(\rho )} \ge H_{\alpha }(A|RE)_{\rho } + \lim _{n \rightarrow \infty } \frac{1}{n} \inf _{\omega _{R^n E^n {{\tilde{E}}}^n}} H_{\alpha }((A')^n|(E')^n {{\tilde{E}}}^n)_{\mathcal {M}^{\otimes n}(\omega )} \,. \end{aligned}$$

This chain rule resembles Lemma 1.2, but is significantly weaker and cannot be used to prove a useful entropy accumulation theorem. The reason for this is twofold:

(i) Equation (1.6) provides a lower bound in terms of $H_{\alpha }(A|RE)$, not $H_{\alpha }(A|E)$. The additional conditioning on the *R*-system can drastically lower the entropy: for example, in a device-independent scenario, *R* would describe the internal memory of the device. Then, Alice’s output *A* contains no entropy when conditioned on the internal memory of the device that produced the output, i.e. $H_{\alpha }(A|RE) = 0$. On the other hand, Alice’s output conditioned only on Eve’s side information *E* may be quite large (and can usually be certified by playing a non-local game), i.e. $H_{\alpha }(A|E) > 0$.
(ii) Equation (1.6) contains the regularised quantity $\lim _{n \rightarrow \infty } \frac{1}{n} \inf _{\omega _{R^n E^n {{\tilde{E}}}^n}} H_{\alpha }((A')^n|(E')^n {{\tilde{E}}}^n)_{\mathcal {M}^{\otimes n}(\omega )}$. Due to the limit $n \rightarrow \infty$, this quantity cannot be computed numerically and therefore the bound in Eq. (1.6) cannot be evaluated for concrete examples.

We now describe how we overcome each of these issues in turn.

(i) We prove a new variant of Uhlmann’s theorem [10], a foundational result in quantum information theory. The original version of Uhlmann’s theorem deals with the case of $\alpha = 1/2$; we show that for $\alpha > 1$, a similar result holds, but an additional regularisation is required. Concretely, we prove that for any states $\rho _{ARE}$ and $\sigma _{AE}$:
  1.7 $$\begin{aligned} \lim _{k \rightarrow \infty } \frac{1}{k} \inf _{\begin{array}{c} {\hat{\sigma }}_{A^k R^k E^k} \\ {\text { s.t. }}{\hat{\sigma }}_{A^k E^k} = \sigma _{AE}^{\otimes k} \end{array}} D_\alpha \! \left( \rho _{ARE}^{\otimes k} \, \big \Vert \, {\hat{\sigma }}_{A^k R^k E^k} \right) = D_\alpha \! \left( \rho _{AE} \, \big \Vert \, \sigma _{AE} \right) \,. \end{aligned}$$
  The proof of this result relies heavily on the spectral pinching technique [11, 12] and we refer to Lemma 3.3 for details as well as a non-asymptotic statement with explicit error bounds. We make use of this extended Uhlmann’s theorem as follows: for the case we are interested in, the map $\mathcal {F}$ in Eq. (1.5) satisfies a non-signalling condition. We can show that this condition implies that for any state $\hat{\sigma }_{A^k R^k E^k} {\text { s.t. }}{\hat{\sigma }}_{A^k E^k} = \sigma _{AE}^{\otimes k}$:
  $$\begin{aligned} D_\alpha \! \left( \mathcal {M}(\rho ) \, \big \Vert \, \mathcal {F}(\sigma ) \right) = \frac{1}{k} D_\alpha \! \left( \mathcal {M}^{\otimes k}(\rho _{ARE}^{\otimes k}) \, \big \Vert \, \mathcal {F}^{\otimes k}({\hat{\sigma }}_{A^k R^k E^k}) \right) \,. \end{aligned}$$
  Applying Eq. (1.5) to the r.h.s. of this equality results in a bound that contains $D_\alpha \! \left( \rho _{ARE}^{\otimes k} \, \big \Vert \, {\hat{\sigma }}_{A^k R^k E^k} \right)$. We can now minimise over all states ${\hat{\sigma }}_{A^k R^k E^k} {\text { s.t. }}{\hat{\sigma }}_{A^k E^k} = \sigma _{AE}^{\otimes k}$ and take the limit $k \rightarrow \infty$. Then, Eq. (1.7) allows us to drop the *R*-system. Therefore, under the non-signalling condition on $\mathcal {F}$, we obtain the following improved chain rule for the sandwiched Rènyi divergence, which might be of independent interest:
  $$\begin{aligned} D_\alpha \! \left( \mathcal {M}(\rho ) \, \big \Vert \, \mathcal {F}(\sigma ) \right) \le D_\alpha \! \left( \rho _{AE} \, \big \Vert \, \sigma _{AE} \right) + \lim _{n \rightarrow \infty } \frac{1}{n} \sup _{\omega _{R^n E^n \tilde{E}^n}}D_\alpha \! \left( \mathcal {M}^{\otimes n}(\omega ) \, \big \Vert \, \mathcal {F}^{\otimes n}(\omega ) \right) \,. \end{aligned}$$
  Using this chain rule, we can show that Eq. (1.6) still holds if $H_{\alpha }(A|RE)$ is replaced by $H_{\alpha }(A|E)$.
(ii) To remove the need for a regularisation in Eq. (1.6), we show that due to the permutation-invariance of $\mathcal {M}^{\otimes n}$ and $\mathcal {F}^{\otimes n}$, for $\alpha > 1$ and $n \rightarrow \infty$ one can replace the optimization over $\omega _{R^n E^n {{\tilde{E}}}^n}$ with a fixed input state, namely the projector onto the symmetric subspace of $R^n E^n {{\tilde{E}}}^n$. For this replacement, one incurs a small loss in $\alpha$, replacing it by $\frac{1}{2 - \alpha }$ (which is only slightly larger than $\alpha$ in the typical regime where $\alpha$ is close to 1). The projector onto the symmetric subspace has a known representation as a mixture of tensor product states [13]. Combining these two steps, we show that the optimization over $\omega _{R^n E^n \tilde{E}^n}$ can be restricted to tensor product states, which means that the regularisation in Eq. (1.6) can be removed (see Sect. 3.2 for details):
  $$\begin{aligned} \lim _{n \rightarrow \infty } \frac{1}{n} \inf _{\omega _{R^n E^n {{\tilde{E}}}^n}} H_{\alpha }((A')^n|(E')^n {{\tilde{E}}}^n)_{\mathcal {M}^{\otimes n}(\omega )} \ge \inf _{\omega _{RE{{\tilde{E}}}}} H_{\frac{1}{2-\alpha }}(A'|E' \tilde{E})_{\mathcal {M}(\omega )} \,. \end{aligned}$$

Combining these results yields Lemma 1.2 and, as a result, our generalised EAT.

*Sample application: blind randomness expansion.* The main advantage of the generalised EAT over previous results is its broader applicability. For example, as demonstrated in [7], the generalised EAT can be used to prove the security of prepare-and-measure QKD protocols, which is of immediate practical relevance, and can also simplify the analysis of entanglement-based QKD protocols as discussed in Sect. 5.2. Here, we focus on the application of our generalised EAT to mistrustful device-independent (DI) cryptography. In mistrustful DI cryptography, multiple parties each use a quantum device to execute a protocol with one another. Each party trusts neither its quantum device nor the other parties in the protocol. Hence, from the point of view of one party, say Alice, all the remaining parties in the protocol are collectively treated as an adversary Eve, who may also have prepared Alice’s untrusted device.

While the original EAT could be used to analyse DI protocols in which the parties trust each other, e.g. DIQKD [14], the setting of mistrustful DI cryptography is significantly harder to analyse because the adversary Eve actively participates in the protocol and may update her side information during the protocol in arbitrary ways. Analysing such protocols requires the more general model of side information we deal with in this paper. As a concrete example for mistrustful DI cryptography, we consider blind randomness expansion, a primitive introduced in [15]. Previous work [15, 16] could only analyse blind randomness expansion under the i.i.d. assumption. Here, we give the first proof that blind randomness expansion is possible for general adversaries. The proof is a straightforward application of our generalised EAT and briefly sketched below; we refer to Sect. 5.1 for a detailed treatment.

In blind randomness expansion, Alice receives an untrusted quantum device from the adversary Eve. Alice then plays a non-local game, e.g. the CHSH game, with this device and Eve, and wants to extract certified randomness from her outputs of the non-local game, i.e. we need to show that Alice’s outputs contain a certain amount of min-entropy conditioned on Eve’s side information. Concretely, in each round of the protocol Alice samples inputs *x* and *y* for the non-local game, inputs *x* into her device to receive outcome *a*, and sends *y* to Eve to receive outcome *b*; Alice then checks whether (*x*, *y*, *a*, *b*) satisfies the winning condition of the non-local game. For comparison, recall that in standard DI randomness expansion [17–21], Alice receives two devices from Eve and uses them to play the non-local game. This means that in standard DI randomness expansion, Eve never learns any of the inputs and outputs of the game. In contrast, in blind randomness expansion Eve learns one of the inputs, *y*, and is free to choose one of the outputs, *b*, herself. Hence, Eve can choose the output *b* based on past side information and update her side information in each round of the protocol using the values of *y* and *b*.

To analyse such a protocol, we use the setting of Theorem 1.1, with $A_i$ representing the output of Alice’s device *D* from the non-local game in the *i*-th round, $R_i$ the internal memory of *D* after the *i*-th round, and $E_i$ Eve’s side information after the *i*-th round, which can be generated arbitrarily from entanglement shared between Eve and *D* at the start of the protocol and information Eve gathered during the first *i* rounds of the protocol. The map $\mathcal {M}_i$ describes one round of the protocol, and because Alice’s device and Eve cannot communicate during the protocol it is easy to show that the non-signalling condition from Theorem 1.1 is satisfied. Therefore, we can apply Theorem 1.1 to lower-bound Alice’s conditional min-entropy $H_\text {min}(A^n|E_n)$ in terms of the single-round quantities $\inf _{\omega } H(A_i|E_i {{\tilde{E}}}_{i-1} )_{\mathcal {M}_i(\omega )}$.^7^ This single-round quantity corresponds to the i.i.d. scenario, i.e. the generalised EAT has reduced the problem of showing blind randomness expansion against general adversaries to the (much simpler) problem of showing it against i.i.d. adversaries. The quantity $\inf _{\omega } H(A_i|E_i {{\tilde{E}}}_{i-1} )_{\mathcal {M}_i(\omega )}$ can be computed using a general numerical technique [22], and for certain classes of non-local games it may also be possible to find an analytical lower bound using ideas from [15, 16]. Inserting the single-round bound, we obtain a lower bound on $H_\text {min}(A^n|E_n)$ that scales linearly with *n*, showing that blind randomness expansion is possible against general adversaries. We also note that as explained in [15], this result immediately implies that *unbounded* randomness expansion is possible with only three devices, whereas previous works required four devices [21, 23, 24].

*Future work* In this work, we have developed a new information-theoretic tool, the generalised EAT. The generalised EAT deals with a more general model of side information than previous techniques and is therefore more broadly and easily applicable. In particular, our generalised EAT can be used to analyse mistrustful DI cryptography. We have demonstrated this by giving the first proof of blind randomness expansion against general adversaries. We expect that the generalised EAT could similarly be used for other protocols such as two-party cryptography in the noisy storage model [25] or certified deletion [16, 26, 27]. In addition to mistrustful DI cryptography, our result can also be used to give new proofs for device-dependent QKD, as demonstrated in Sect. 5.2 and [7], and is applicable to proving the security of commercial quantum random number generators, which typically have correlations between rounds due to experimental imperfections [28].

Beyond cryptography, the generalised EAT is useful whenever one is interested in bounding the min-entropy of a large system that can be decomposed in a sequential way. Such problems are abundant in physics. For example, the dynamics of an open quantum system can be described in terms of interactions that take place sequentially with different parts of the system’s environment [29]. In quantum thermodynamics, such a description is commonly employed to model the thermalisation of a system that is brought in contact with a thermal bath. For a lack of techniques, the entropy flow during a thermalisation process of this type is usually quantified in terms of von Neumann entropy rather than the operationally more relevant smooth min- and max-entropies [30]. The generalised EAT may be used to remedy this situation. A similar situation arises in quantum gravity, where smooth entropies play a role in the study of black holes [31].

In a different direction, one can also try to further improve the generalised EAT itself. Compared to the original EAT [1], our generalised EAT features a more general model of side information and a weaker condition on the relation between different rounds, replacing the Markov condition of [1] with our weaker non-signalling condition in Eq. (1.2). It is natural to ask whether a further step in this direction is possible: while the model of side information we consider is fully general, it may be possible to replace the non-signalling condition with a weaker requirement. We have argued above that our non-signalling condition appears to be the most general way of stating the requirement that future side information does not reveal information about past outputs, which seems necessary for an EAT-like theorem.^8^ It would be interesting to formalise this intuition and see whether our theorem is provably “tight” in terms of the conditions placed on the sequential process. Furthermore, it might be possible to improve the way the statistical condition in Theorem 4.3 is dealt with in the proof, e.g. using ideas from [33, 34].

Finally, one could attempt to extend entropy accumulation from conditional entropies to relative entropies. Such a *relative entropy accumulation theorem* (REAT) would be the following statement: for two sequences of channels $\{\mathcal {E}_1, \dots , \mathcal {E}_n\}$ and $\{\mathcal {F}_1, \dots , \mathcal {F}_n\}$ (where $\mathcal {F}_i$ need not necessarily be trace-preserving), and $\varepsilon > 0$,

$$\begin{aligned} D_{\text {max}}^\varepsilon \! \left( \mathcal {E}_n \circ \dots \circ \mathcal {E}_1 \, \big \Vert \, \mathcal {F}_n \circ \dots \circ \mathcal {F}_1 \right) {\mathop {\le }\limits ^{?}} \sum _{i=1}^n D^\text {reg} \! \left( \mathcal {E}_i \, \big \Vert \, \mathcal {F}_i \right) + O(\sqrt{n}) \,. \end{aligned}$$

Here, $D_{\text {max}}^\varepsilon$ is the $\varepsilon$-smooth max-relative entropy [11] and we used the (regularised) channel divergences defined in Definition 2.5. The key technical challenge in proving this result is to show that the regularised channel divergence $D^\text {reg}_\alpha \! \left( \mathcal {E}_i \, \big \Vert \, \mathcal {F}_i \right)$ is continuous in $\alpha$ at $\alpha = 1$, which is an important technical open question. If one had such a continuity statement and the maps $\mathcal {F}_i$ additionally satisfied a non-signalling condition (which is not required for the statement above), one could also use our Theorem 3.1 to derive a more general REAT, which would imply our generalised EAT.

## Preliminaries

### Notation

Throughout this paper, we restrict ourselves to finite-dimensional Hilbert spaces. The set of positive semidefinite operators on a quantum system *A* (with associated Hilbert space $\mathcal {H}_A$) is denoted by ${{\,\textrm{Pos}\,}}(A)$. The set of quantum states is given by $\text {S}(A) = \{\rho \in {{\,\textrm{Pos}\,}}(A) \, | \, \text{ Tr }\!\left[ \rho \right] = 1\}$. The set of completely positive maps from linear operators on *A* to linear operators on $A'$ is denoted by $\text {CP}(A, A')$. If such a map is additionally trace preserving, we call it a quantum channel and denote the set of such maps by $\text {CPTP}(A, A')$. The identity channel on system *A* is denoted as $\text {id}_A$. The spectral norm is denoted by $\left\Vert \cdot \right\Vert _{\infty }$.

A cq-state is a quantum state $\rho \in \text {S}(XA)$ on a *classical* system *X* (with alphabet $\mathcal {X}$) and a quantum system *A*, i.e. a state that can be written as

$$\begin{aligned} \rho _{XA} = \sum _{x \in \mathcal {X}} |x\rangle \!\langle x| \otimes \rho _{A, x} \end{aligned}$$

for subnormalised $\rho _{A, x} \in {{\,\textrm{Pos}\,}}(A)$. For $\Omega \subset \mathcal {X}$, we define the conditional state

$$\begin{aligned} \rho _{XA|\Omega } = \frac{1}{\textrm{Pr}_{\rho }\!\left[ \Omega \right] } \sum _{x \in \Omega } |x\rangle \!\langle x| \otimes \rho _{A, x} \,, \quad \text {where } \; \textrm{Pr}_{\rho }\!\left[ \Omega \right] {:}{=}\sum _{x \in \Omega } \text{ Tr }\!\left[ \rho _{A, x} \right] \,. \end{aligned}$$

If $\Omega = \{x\}$, we also write $\rho _{XA|x}$ for $\rho _{XA|\Omega }$.

### Rényi divergence and entropy

We will make extensive use of the sandwiched Rényi divergence [35, 36] and quantities associated with it, namely Rényi entropies and channel divergences. We recall the relevant definitions here.

#### Definition 2.1

(Rényi divergence). For $\rho \in \text {S}(A)$, $\sigma \in {{\,\textrm{Pos}\,}}(A)$, and $\alpha \in [1/2,1) \cup (1,\infty )$ the (sandwiched) Rényi divergence is defined as

$$\begin{aligned} D_\alpha \! \left( \rho \, \big \Vert \, \sigma \right) {:}{=}\frac{1}{\alpha -1} \log \text{ Tr }\!\left[ \Big (\sigma ^{\frac{1-\alpha }{2 \alpha }} \rho \sigma ^{\frac{1-\alpha }{2 \alpha }}\Big )^{\alpha } \right] \end{aligned}$$

for ${{\,\mathrm{\text {supp}}\,}}(\rho ) \subseteq {{\,\mathrm{\text {supp}}\,}}(\sigma )$, and $+\infty$ otherwise.

From the Rényi divergence, one can define the conditional Rényi entropies as follows (see [11] for more details).

#### Definition 2.2

*(Conditional Rényi entropy)*. For a bipartite state $\rho _{AB} \in \text {S}(AB)$ and $\alpha \in [1/2,1) \cup (1,\infty )$, we define the following two conditional Rényi entropies:

From the definition it is clear that . Importantly, a relation for the other direction also holds.

#### Lemma 2.3

([11, Corollary 5.3]). For $\rho _{AB} \in \text {S}(AB)$ and $\alpha \in (1,2)$:

In the limit $\alpha \rightarrow 1$ the sandwiched Rényi divergence converges to the relative entropy:

$$\begin{aligned} \lim _{\alpha \rightarrow 1} D_\alpha \! \left( \rho \, \big \Vert \, \sigma \right) = D(\rho \Vert \sigma ) = \text{ Tr }\!\left[ \rho (\log \rho - \log \sigma ) \right] \,. \end{aligned}$$

Accordingly, the conditional Rényi entropy converges to the conditional von Neumann entropy:

$$\begin{aligned} \lim _{\alpha \rightarrow 1} H_{\alpha }(A|B)_{\rho } = H(A|B)_\rho = H(AB)_\rho - H(B)_\rho = - \text{ Tr }\!\left[ \rho _{AB} \log \rho _{AB} \right] + \text{ Tr }\!\left[ \rho _{B} \log \rho _{B} \right] \,. \end{aligned}$$

Conversely, in the limit $\alpha \rightarrow \infty$, the Rényi entropy  converges to the min-entropy. We will make use of a smoothed version of the min-entropy, which is defined as follows [2].

#### Definition 2.4

*(Smoothed min-entropy).* For $\rho _{AB} \in \text {S}(AB)$ and $\varepsilon \in [0,1]$, the $\varepsilon$-smoothed min-entropy of *A* conditioned on *B* is

$$\begin{aligned} H_{\min }^\varepsilon (A|B)_{\rho } = - \log \inf _{{\tilde{\rho }}_{AB} \in \mathcal {B}_{\varepsilon }(\rho _{AB})} \inf _{\sigma _{B} \in \text {S}(B)} \left\Vert \sigma _B^{-\frac{1}{2}} {\tilde{\rho }}_{AB} \sigma _B^{-\frac{1}{2}} \right\Vert _{\infty } \, , \end{aligned}$$

where $\left\Vert \cdot \right\Vert _{\infty }$ denotes the spectral norm and $\mathcal {B}_{\varepsilon }(\rho _{AB})$ is the $\varepsilon$-ball around $\rho _{AB}$ in term of the purified distance [11].

Finally, we can extend the definition of the Rényi divergence from states to channels. The resulting quantity, the channel divergence (and its regularised version), will play an important role in the rest of the manuscript.

#### Definition 2.5

*(Channel divergence).* For $\mathcal {E}\in \text {CPTP}(A, A')$, $\mathcal {F}\in \text {CP}(A, A')$, and $\alpha \in [1/2,1) \cup (1,\infty )$, the (stabilised) channel divergence^9^ is defined as

2.1 $$\begin{aligned} D_\alpha \! \left( \mathcal {E} \, \big \Vert \, \mathcal {F} \right) = \sup _{\omega \in \text {S}(A{{\tilde{A}}})} D_\alpha \! \left( \mathcal {E}(\omega ) \, \big \Vert \, \mathcal {F}(\omega ) \right) \,, \end{aligned}$$

where without loss of generality ${{\tilde{A}}} \equiv A$. The regularised channel divergence is defined as

$$\begin{aligned} D^\text {reg}_\alpha \! \left( \mathcal {E} \, \big \Vert \, \mathcal {F} \right) {:}{=}\lim _{n \rightarrow \infty } \frac{1}{n} D_\alpha \! \left( \mathcal {E}^{\otimes n} \, \big \Vert \, \mathcal {F}^{\otimes n} \right) = \sup _n \frac{1}{n} D_\alpha \! \left( \mathcal {E}^{\otimes n} \, \big \Vert \, \mathcal {F}^{\otimes n} \right) \,. \end{aligned}$$

We note that the channel divergence is in general not additive under the tensor product [37, Proposition 3.1], so the regularised channel divergence can be strictly larger that the non-regularised one, i.e., $D^\text {reg}_\alpha \! \left( \mathcal {E} \, \big \Vert \, \mathcal {F} \right) > D_\alpha \! \left( \mathcal {E} \, \big \Vert \, \mathcal {F} \right)$. The regularised channel divergence, however, does satisfy an additivity property:

2.2 $$\begin{aligned}&D^\text {reg}_\alpha \! \left( \mathcal {E}^{\otimes k} \, \big \Vert \, \mathcal {F}^{\otimes k} \right) \nonumber \\&= \lim _{n \rightarrow \infty } \frac{1}{n} D_\alpha \! \left( \mathcal {E}^{\otimes k n} \, \big \Vert \, \mathcal {F}^{\otimes k n} \right) = k \lim _{n \rightarrow \infty } \frac{1}{n'} D_\alpha \! \left( \mathcal {E}^{\otimes n'} \, \big \Vert \, \mathcal {F}^{\otimes n'} \right) = k \, D^\text {reg}_\alpha \! \left( \mathcal {E} \, \big \Vert \, \mathcal {F} \right) \,, \end{aligned}$$

where we switched to the index $n' = k n$ for the second equality.

### Spectral pinching

A key technical tool in our proof will be the use of spectral pinching maps [38], which are defined as follows (see [12, Chapter 3] for a more detailed introduction).

#### Definition 2.6

*(Spectral pinching map).* Let $\rho \in {{\,\textrm{Pos}\,}}(A)$ with spectral decomposition $\rho =\sum _{\lambda } \lambda P_{\lambda }$, where $\lambda \in \text {Spec}(\rho ) \subset \mathbb {R}_{\ge 0}$ are the distinct eigenvalues of $\rho$ and $P_{\lambda }$ are mutually orthogonal projectors. The *(spectral) pinching map* $\mathcal {P}_{\rho } \in \text {CPTP}(A, A)$ associated with $\rho$ is given by

$$\begin{aligned} \mathcal {P}_{\rho }(\omega ) {:}{=}\sum _{\lambda \in \text {Spec}(\rho )} P_\lambda \, \omega \, P_{\lambda } \,. \end{aligned}$$

We will need a few basic properties of pinching maps.

#### Lemma 2.7

(Properties of pinching maps). For any $\rho ,\sigma \in {{\,\textrm{Pos}\,}}(A)$, the following properties hold:

(i) Invariance: $$\mathcal {P}_{\rho }(\rho )=\rho $$ .
(ii) Commutation of pinched state: $[\sigma , \mathcal {P}_{\sigma }(\rho )] = 0$ .
(iii) Pinching inequality: $\mathcal {P}_{\sigma }(\rho ) \ge \frac{1}{|\text {Spec}(\sigma )|}\rho$ .
(iv) Commutation of pinching maps: if $[\rho ,\sigma ]=0$, then $\mathcal {P}_{\rho } \circ \mathcal {P}_{\sigma } = \mathcal {P}_{\sigma } \circ \mathcal {P}_{\rho }$ .
(v) Partial trace: $$ \text{ Tr}_{B}\!\left[ \mathcal {P}_{\rho _A \otimes {\mathbb {1}}_B}(\omega _{AB}) \right] = \mathcal {P}_{\rho _A}(\omega _A) \quad \forall \,\omega _{AB} \in {{\,\textrm{Pos}\,}}(AB)$$.

#### Proof

Properties (i)–(iii) follow from the definition and [3, Chapter 2.6.3] or [12, Lemma 3.5].

For the fourth statement, note that since $[\rho , \sigma ] = 0$, there exists a joint orthonormal eigenbasis $\{|x_i\rangle \}$ of $\rho$ and $\sigma$. Let $P_\lambda$ be the projector onto the eigenspace of $\rho$ with eigenvalue $\lambda$, and $Q_\mu$ the projector onto the eigenspace of $\sigma$ with eigenvalue $\mu$. We can expand

$$\begin{aligned} P_\lambda = \sum _{i {\text { s.t. }}\rho |x_i\rangle = \lambda |x_i\rangle } |x_i\rangle \!\langle x_i| \qquad \text {and} \qquad Q_\mu = \sum _{j {\text { s.t. }}\sigma |x_j\rangle = \mu |x_j\rangle } |x_j\rangle \!\langle x_j| \,. \end{aligned}$$

Since $\{|x_i\rangle \}$ is a family of orthonormal vectors,

$$\begin{aligned} P_\lambda Q_{\mu } = \sum _{\begin{array}{c} i {\text { s.t. }}\rho |x_i\rangle = \lambda |x_i\rangle \\ \;\text { and }\;\sigma |x_i\rangle = \mu |x_i\rangle \end{array}} |x_i\rangle \!\langle x_i| = Q_{\mu } P_\lambda \,, \end{aligned}$$

which implies commutation of the pinching maps.

For the fifth statement, note that if we write $\rho = \sum _{\lambda } \lambda P_\lambda$ with eigenprojectors $P_{\lambda }$, then the set of eigenprojectors of $\rho _A \otimes {\mathbb {1}}_B$ is simply $\{P_\lambda \otimes {\mathbb {1}}_B\}$. Hence,

$$\begin{aligned} \text{ Tr}_{B}\!\left[ \mathcal {P}_{\rho _A \otimes {\mathbb {1}}_B}(\omega _{AB}) \right]&= \sum _{\lambda } \text{ Tr}_{B}\!\left[ P_\lambda \otimes {\mathbb {1}}_B \omega _{AB} P_\lambda \otimes {\mathbb {1}}_B \right] \\&= \sum _{\lambda } P_\lambda \text{ Tr}_{B}\!\left[ \omega _{AB} \right] P_\lambda = \mathcal {P}_{\rho _A}(\omega _A) \,. \end{aligned}$$

$$\square $$

It is often useful to use the pinching map associated with tensor power states, i.e., $\mathcal {P}_{\rho ^{\otimes n}}$. This is because for $\rho \in {{\,\textrm{Pos}\,}}(A)$, the factor $|\text {Spec}(\rho ^{\otimes n})|$ from the pinching inequality (see Lemma 2.7) only scales polynomially in *n* (see e.g. [12, Remark 3.9]):

2.3 $$\begin{aligned} |\text {Spec}(\rho ^{\otimes n})| \le (n+1)^{\dim (A)-1} \,. \end{aligned}$$

In fact, we can show a similar property for all permutation-invariant states, not just tensor product states.

#### Lemma 2.8

Let $\rho \in {{\,\textrm{Pos}\,}}(A^{\otimes n})$ be permutation invariant and denote $d = \dim (A)$. Then

$$\begin{aligned} |\text {Spec}(\rho )| \le (n+d)^{d(d+1)/2} \,. \end{aligned}$$

#### Proof

By Schur-Weyl duality and Schur’s lemma (see e.g. [39, Lemma 0.8 and Theorem 1.10]), since $\rho$ is permutation-invariant, we have

$$\begin{aligned} \rho \cong \bigoplus _{\lambda \in \mathcal {I}_{d, n}} \rho (\lambda )_{Q_\lambda } \otimes {\mathbb {1}}_{P_{\lambda }} \,, \end{aligned}$$

where $\cong$ denotes equality up to unitary conjugation (which leaves the spectrum invariant), $\mathcal {I}_{d, n}$ is the set of Young diagrams with *n* boxes and at most *d* rows, $Q_\lambda$ and $P_\lambda$ are systems whose details need not concern us, and $\rho (\lambda ) \in {{\,\textrm{Pos}\,}}(Q_\lambda )$. From this it is clear that

$$\begin{aligned} |\text {Spec}(\rho )| \le \sum _{\lambda \in \mathcal {I}_{d, n}} |\text {Spec}(\rho (\lambda ))| \le \sum _{\lambda \in \mathcal {I}_{d, n}} \dim (Q_{\lambda }) \,. \end{aligned}$$

It is known that $|\mathcal {I}_{d, n}| \le (n+1)^d$ and $\dim (Q_\lambda ) \le (n+d)^{d(d-1)/2}$ (see e.g. [40, Section 6.2]). Hence

$$\begin{aligned} |\text {Spec}(\rho )| \le (n+1)^d (n+d)^{d(d-1)/2} \le (n+d)^{d(d+1)/2} \,. \end{aligned}$$

$$\square $$

#### Corollary 2.9

Let $\rho , \sigma \in {{\,\textrm{Pos}\,}}(A)$ and $d = \dim (A)$. Then

$$\begin{aligned} |\text {Spec}\!\left( \mathcal {P}_{\rho ^{\otimes n}}(\sigma ^{\otimes n}) \right) | \le (n+d)^{d(d+1)/2} \,. \end{aligned}$$

#### Proof

Note that $\mathcal {P}_{\rho ^{\otimes n}}(\sigma ^{\otimes n})$ is itself not a product state because the eigenprojectors of $\rho ^{\otimes n}$ do not have a product form. However, since every eigenspace of $\rho ^{\otimes n}$ is permutation-invariant, $\mathcal {P}_{\rho ^{\otimes n}}(\sigma ^{\otimes n})$ is permutation-invariant, too, so we can apply Lemma 2.8. $\square$

## Strengthened Chain Rules

One of the crucial properties of entropies are chain rules, which allow us to relate entropies of large composite systems to sums of entropies of the individual subsystems. In this section, we prove two new such chain rules, one for the Rényi divergence (Theorem 3.1, which is a generalisation of [9, Corollary 5.1]) and one for the conditional entropy (Theorem 3.6). The chain rule from Theorem 3.6 is the key ingredient for our generalised EAT, to which we will turn our attention in Sect. 4. Theorem 3.6 plays a similar role for our generalised EAT as [1, Corollary 3.5] does for the original EAT, but while the latter requires a Markov condition, the former does not. As a result, our generalised EAT based on Theorem 3.6 also avoids the Markov condition.

The outline of this section is as follows: we first prove a generalised chain rule for the Rényi divergence (Theorem 3.1). This chain rule contains a regularised channel divergence. As the next step, we show that in the special case of conditional entropies, we can drop the regularisation (Sect. 3.2). This allows us to derive a chain rule for conditional entropies from the chain rule for channels (Sect. 3.3).

### Strengthened chain rule for Rényi divergence

The main result of this section is the following chain rule for the Rényi divergence.

#### Theorem 3.1

Let $\alpha > 1$, $\rho \in \text {S}(A R)$, $\sigma \in {{\,\textrm{Pos}\,}}(A R)$, $\mathcal {E}\in \text {CPTP}(AR, B)$, and $\mathcal {F}\in \text {CP}(AR, B)$. Suppose that there exists $\mathcal {R}\in \text {CP}(A, B)$ such that $\mathcal {F}= \mathcal {R}\circ \text {Tr}_{R}$. Then

3.1 $$\begin{aligned} D_\alpha \! \left( \mathcal {E}(\rho _{AR}) \, \big \Vert \, \mathcal {F}(\sigma _{AR}) \right) \le D_\alpha \! \left( \rho _{A} \, \big \Vert \, \sigma _{A} \right) + D^\text {reg}_\alpha \! \left( \mathcal {E} \, \big \Vert \, \mathcal {F} \right) \,. \end{aligned}$$

This is a stronger version of an existing chain rule due to [9], which we will use in our proof of Theorem 3.1:

#### Lemma 3.2

([9, Corollary 5.1]). Let $\alpha > 1$, $\rho \in \text {S}(A)$, $\sigma \in {{\,\textrm{Pos}\,}}(A)$, $\mathcal {E}\in \text {CPTP}(A, B)$, and $\mathcal {F}\in \text {CP}(A, B)$. Then

3.2 $$\begin{aligned} D_\alpha \! \left( \mathcal {E}(\rho ) \, \big \Vert \, \mathcal {F}(\sigma ) \right) \le D_\alpha \! \left( \rho \, \big \Vert \, \sigma \right) + D^\text {reg}_\alpha \! \left( \mathcal {E} \, \big \Vert \, \mathcal {F} \right) \,. \end{aligned}$$

The difference between Theorem 3.1 and Lemma 3.2 is that on the r.h.s. of Eq. (3.1), we only have the divergence $D_\alpha \! \left( \rho _{A} \, \big \Vert \, \sigma _{A} \right)$ between the two reduced states on system *A*. In contrast, if we used Eq. (3.2) with systems *AR*, then we would get the divergence $D_\alpha \! \left( \rho _{AR} \, \big \Vert \, \sigma _{AR} \right)$ between the full states. In particular, the weaker Lemma 3.2 can easily be recovered from Theorem 3.1 by taking the system *R* to be trivial, in which case the condition $\mathcal {F}= \mathcal {R}\circ \text {Tr}_{R}$ becomes trivial, too.

While the difference between Theorem 3.1 and Lemma 3.2 may look minor at first sight, the two chain rules can give considerably different results: in general, the data processing inequality ensures that $D_\alpha \! \left( \rho _{A} \, \big \Vert \, \sigma _{A} \right) \le D_\alpha \! \left( \rho _{AR} \, \big \Vert \, \sigma _{AR} \right)$, but the gap between the two quantities can be significant, i.e., there exist states for which $D_\alpha \! \left( \rho _{A} \, \big \Vert \, \sigma _{A} \right) \ll D_\alpha \! \left( \rho _{AR} \, \big \Vert \, \sigma _{AR} \right)$. In such cases, Theorem 3.1 yields a significantly tighter bound. This turns out to be crucial if we want to apply this chain rule repeatedly to get an EAT.

We also note that the statement of Theorem 3.1 is known to be correct also for $\alpha =1$ [37, Theorem 3.5]. However, this requires a separate proof and does not follow from Theorem 3.1 as it is currently not known whether the function $\alpha \mapsto D^\text {reg}_\alpha \! \left( \mathcal {E} \, \big \Vert \, \mathcal {F} \right)$ is continuous in the limit $\alpha \searrow 1$.^10^

We now turn to the proof of Theorem 3.1. The key question for the proof is the following: given states $\rho _{AR}$ and $\sigma _{A}$, does there exist an extension $\sigma _{AR}$ of $\sigma _A$ such that $D_\alpha \! \left( \rho _A \, \big \Vert \, \sigma _A \right) = D_\alpha \! \left( \rho _{AR} \, \big \Vert \, \sigma _{AR} \right)$? For the special case of $\alpha = 1/2$, an affirmative answer is given by Uhlmann’s theorem [10] (see also [11, Corollary 3.14]). This also holds for $\alpha = \infty$, but not in general for $\alpha \ge 1$ as discussed in Sect. B. The following lemma shows that a similar property still holds for $\alpha > 1$ on a regularised level.

#### Lemma 3.3

Consider quantum systems *A* and *R* with $d = \dim (A)$. For $n \in \mathbb {N}$, we define $A^n = A_1 \dots A_n$, where $A_i$ are copies of the system *A*, and likewise $R^n = R_1 \dots R_n$. Then for $\rho \in \text {S}(AR)$, $\sigma \in {{\,\textrm{Pos}\,}}(A)$, and $\alpha > 1$ we have

$$\begin{aligned}&D_\alpha \! \left( \rho _A \, \big \Vert \, \sigma _A \right) \le \inf _{{\hat{\sigma }}_{A^n R^n} {\text { s.t. }}\hat{\sigma }_{A^n} = \sigma _A^{\otimes n}} \frac{1}{n} D_\alpha \! \left( \rho _{AR}^{\otimes n} \, \big \Vert \, {\hat{\sigma }}_{A^n R^n} \right) \\&\quad \le D_\alpha \! \left( \rho _A \, \big \Vert \, \sigma _A \right) + \frac{\alpha }{\alpha -1} \frac{d(d+1) \log (n+d)}{n} \, . \end{aligned}$$

#### Proof

The inequality

$$\begin{aligned} D_\alpha \! \left( \rho _A \, \big \Vert \, \sigma _A \right) \le \inf _{{\hat{\sigma }}_{A^n R^n} {\text { s.t. }}\hat{\sigma }_{A^n} = \sigma _A^{\otimes n}} \frac{1}{n} D_\alpha \! \left( \rho _{AR}^{\otimes n} \, \big \Vert \, {\hat{\sigma }}_{A^n R^n} \right) \end{aligned}$$

follows directly from the data processing inequality for taking the partial trace over $R^n$, and additivity of $D_{\alpha }$ under tensor product [11].

For the other direction, we consider *n*-fold tensor copies of $\rho _{AR}$ and $\sigma _{A}$, which we denote by $\rho _{A^n R^n} = \rho _{A_1 R_1} \otimes \dots \otimes \rho _{A_n R_n}$ and $\sigma _{A^n} = \sigma _{A_1} \otimes \dots \otimes \sigma _{A_n}$. We define the following two pinched states

3.3 $$\begin{aligned} \rho '_{A^n R^n} = \mathcal {P}_{\sigma _{A^n} \otimes {\mathbb {1}}_{R^n}}(\rho _{A^n R^n}) \qquad \text {and} \qquad {\hat{\rho }}_{A^n R^n} = \mathcal {P}_{\rho '_{A^n} \otimes {\mathbb {1}}_{R^n}}(\rho '_{A^n R^n}) \,. \end{aligned}$$

By definition of ${\hat{\rho }}_{A^n R^n}$ and using the pinching inequality (see Lemma 2.7(iii)) twice, we have

$$\begin{aligned} \rho _{A^n R^n} \le |\text {Spec}(\sigma _{A^n})| |\text {Spec}(\rho '_{A^n})| \; {\hat{\rho }}_{A^n R^n} \,. \end{aligned}$$

Using the operator monotonicity of the sandwiched Rényi divergence in the first argument [11] we find for any state ${\hat{\sigma }}_{A^n R^n}$

3.4 $$\begin{aligned} \frac{1}{n} D_\alpha \! \left( \rho _{AR}^{\otimes n} \, \big \Vert \, {\hat{\sigma }}_{A^nR^n} \right) \le \frac{1}{n} D_\alpha \! \left( {\hat{\rho }}_{A^nR^n} \, \big \Vert \, {\hat{\sigma }}_{A^nR^n} \right) + \frac{1}{n} \frac{\alpha }{\alpha -1} \eta (n) \,, \end{aligned}$$

with the error term

$$\begin{aligned} \eta (n) = \log |\text {Spec}(\sigma _{A^n})| + \log |\text {Spec}(\rho '_{A^n})| \,. \end{aligned}$$

To prove the lemma, we now need to bound the error term $\eta (n)$ and construct a specific choice for ${\hat{\sigma }}_{A^nR^n}$ for which ${\hat{\sigma }}_{A^n} = \sigma _A^{\otimes n}$ and $\frac{1}{n} D_\alpha \! \left( {\hat{\rho }}_{A^nR^n} \, \big \Vert \, {\hat{\sigma }}_{A^nR^n} \right) \le D_\alpha \! \left( \rho _A \, \big \Vert \, \sigma _A \right)$. We first bound $\eta (n)$. Since $\sigma _{A^n} = \sigma _A^{\otimes n}$, we have from Eq. (2.3) that $|\text {Spec}(\sigma _{A^n})| \le (n+1)^{d-1}$, where $d = \dim (A)$. To bound $|\text {Spec}(\rho '_{A^n})|$, we note that by Eq. (3.3) and Lemma 2.7(v)

3.5 $$\begin{aligned} \rho '_{A^n} = \text{ Tr}_{R^n}\!\left[ \mathcal {P}_{\sigma _{A^n} \otimes {\mathbb {1}}_{R^n}}(\rho _{A^n R^n}) \right] = \mathcal {P}_{\sigma _{A^n}}(\rho _{A^n}) = \mathcal {P}_{\sigma _{A}^{\otimes n}}(\rho _{A}^{\otimes n}) \,. \end{aligned}$$

We can therefore use Lemma 2.9 to obtain $|\text {Spec}(\rho '_{A^n})| \le (n+d)^{d(d+1)/2}$. Hence,

3.6 $$\begin{aligned} \eta (n) \le d(d+1) \log (n+d) \,. \end{aligned}$$

It thus remains to construct ${\hat{\sigma }}_{A^n R^n}$ satisfying the properties mentioned above. To do so we first establish a number of commutation statements.

(i) From Lemma 2.7(ii) we have that $[{\hat{\rho }}_{A^n R^n}, \rho '_{A^n} \otimes {\mathbb {1}}_{R^n}] = 0$. Recalling the definition of $\rho '$ from Eq. (3.3), we get
  3.7 $$\begin{aligned} \hat{\rho }_{A^n} = \text{ Tr}_{R^n}\!\left[ \mathcal {P}_{\rho '_{A^n} \otimes {\mathbb {1}}_{R^n}}(\rho '_{A^n R^n}) \right] = \mathcal {P}_{\rho '_{A^n}} (\rho '_{A^n}) = \rho '_{A^n} \,, \end{aligned}$$
  where the final step uses Lemma 2.7(i). As a result we find
  3.8 $$\begin{aligned} [{\hat{\rho }}_{A^n R^n}, {\hat{\rho }}_{A^n} \otimes {\mathbb {1}}_{R^n}] = 0 \,. \end{aligned}$$
(ii) From Lemma 2.7(ii) we have that $[\rho '_{A^n R^n}, \sigma _{A^n} \otimes {\mathbb {1}}_{R^n}] = 0$. Taking the partial trace over $R^n$, this implies $[\rho '_{A^n}, \sigma _{A^n}] = 0$, so by Lemma 2.7(iv) and Eq. (3.3)
  $$\begin{aligned} {\hat{\rho }}_{A^n R^n} = \mathcal {P}_{\rho '_{A^n} \otimes {\mathbb {1}}_{R^n}}\left( \mathcal {P}_{\sigma _{A^n} \otimes {\mathbb {1}}_{R^n}}(\rho _{A^n R^n}) \right) = \mathcal {P}_{\sigma _{A^n} \otimes {\mathbb {1}}_{R^n}}\left( \mathcal {P}_{\rho '_{A^n} \otimes {\mathbb {1}}_{R^n}}(\rho _{A^n R^n}) \right) \,. \end{aligned}$$
  Therefore, by Lemma 2.7(ii),
  3.9 $$\begin{aligned} [{\hat{\rho }}_{A^n R^n}, \sigma _{A^n} \otimes {\mathbb {1}}_{R^n}] = 0 \,. \end{aligned}$$
(iii) Taking the partial trace over $R^n$ in Eq. (3.9), we get
  3.10 $$\begin{aligned} [{\hat{\rho }}_{A^n}, \sigma _{A^n}] = 0 \,. \end{aligned}$$

Having established these commutation relations, we define $\mathcal {T}\in \text {CPTP}(A^n, A^n R^n)$ by^11^

$$\begin{aligned} \mathcal {T}(\omega _{A^n}) = {\hat{\rho }}_{A^n R^n}^{1/2} {\hat{\rho }}_{A^n}^{-1/2} \omega _{A^n} {\hat{\rho }}_{A^n}^{-1/2} {\hat{\rho }}_{A^n R^n}^{1/2}\,. \end{aligned}$$

By construction,

3.11 $$\begin{aligned} \mathcal {T}({\hat{\rho }}_{A^n}) = {\hat{\rho }}_{A^n R^n} \,. \end{aligned}$$

We define

3.12 $$\begin{aligned} \hat{\sigma }_{A^n R^n} = \mathcal {T}(\sigma _{A^n}) \,. \end{aligned}$$

To see that this is a valid choice of ${\hat{\sigma }}$, i.e., that $\hat{\sigma }_{A^n} = \sigma _{A^n} = \sigma _A^{\otimes n}$, we use Eqs. (3.8), (3.9) and (3.10) to find

$$\begin{aligned} \hat{\sigma }_{A^n} = \text{ Tr}_{R^n}\!\left[ {\hat{\rho }}_{A^n R^n}^{1/2} {\hat{\rho }}_{A^n}^{-1/2} \sigma _{A^n} {\hat{\rho }}_{A^n}^{-1/2} {\hat{\rho }}_{A^n R^n}^{1/2} \right] = \text{ Tr}_{R^n}\!\left[ {\hat{\rho }}_{A^n R^n} {\hat{\rho }}_{A^n}^{-1} \sigma _{A^n} \right] = \sigma _{A^n} \,. \end{aligned}$$

Using Eqs. (3.11) and (3.12) followed by the data processing inequality [11], we obtain

3.13 $$\begin{aligned} \frac{1}{n} D_\alpha \! \left( {\hat{\rho }}_{A^n R^n} \, \big \Vert \, {\hat{\sigma }}_{A^n R^n} \right) = \frac{1}{n} D_\alpha \! \left( \mathcal {T}({\hat{\rho }}_{A^n}) \, \big \Vert \, \mathcal {T}(\sigma _{A^n}) \right) \le \frac{1}{n} D_\alpha \! \left( {\hat{\rho }}_{A^n} \, \big \Vert \, \sigma _{A^n} \right) \,. \end{aligned}$$

By Eqs. (3.7) and (3.3) we have ${\hat{\rho }}_{A^n} = \rho '_{A^n} = \mathcal {P}_{\sigma _{A^n}}(\rho _{A^n})$. Therefore, continuing from Eq. (3.13) and using $\sigma _{A^n} = \mathcal {P}_{\sigma _{A^n}}(\sigma _{A^n})$ followed by the data processing inequality gives

$$\begin{aligned} \frac{1}{n} D_\alpha \! \left( {\hat{\rho }}_{A^n R^n} \, \big \Vert \, {\hat{\sigma }}_{A^n R^n} \right) \le \frac{1}{n} D_\alpha \! \left( \rho _{A^n} \, \big \Vert \, \sigma _{A^n} \right) = \frac{1}{n} D_\alpha \! \left( \rho _{A}^{\otimes n} \, \big \Vert \, \sigma _{A}^{\otimes n} \right) = D_\alpha \! \left( \rho _{A} \, \big \Vert \, \sigma _{A} \right) \,. \end{aligned}$$

Inserting this and our error bound from Eq. (3.6) into Eq. (3.4) proves the desired statement. $\square$

With this, we can now prove Theorem 3.1.

#### Proof of Theorem 3.1

Because $D_{\alpha }$ is additive under tensor products, for any $n \in \mathbb {N}$ we have

3.14 $$\begin{aligned} D_\alpha \! \left( \mathcal {E}(\rho _{AR}) \, \big \Vert \, \mathcal {F}(\sigma _{AR}) \right)&= \frac{1}{n} D_\alpha \! \left( \mathcal {E}^{\otimes n}(\rho _{AR}^{\otimes n}) \, \big \Vert \, \mathcal {F}^{\otimes n}(\sigma _{AR}^{\otimes n}) \right) \nonumber \\&= \inf _{{\hat{\sigma }}_{A^n R^n} {\text { s.t. }}{\hat{\sigma }}_{A^n} = \sigma _A^{\otimes n}} \frac{1}{n} D_\alpha \! \left( \mathcal {E}^{\otimes n}(\rho _{AR}^{\otimes n}) \, \big \Vert \, \mathcal {F}^{\otimes n}({\hat{\sigma }}_{A^nR^n}) \right) \,, \end{aligned}$$

where the second equality holds because $\mathcal {F}= \mathcal {R}\circ \text {Tr}_{R}$, so $\mathcal {F}^{\otimes n}(\sigma _{AR}^{\otimes n}) = \mathcal {F}^{\otimes n}({\hat{\sigma }}_{A^nR^n})$ for any $\hat{\sigma }_{A^nR^n}$ that satisfies ${\hat{\sigma }}_{A^n} = \sigma _A^{\otimes n}$. From the chain rule in Lemma 3.2 we get that for any $\hat{\sigma }_{A^nR^n}$:

$$\begin{aligned} \frac{1}{n} D_\alpha \! \left( \mathcal {E}^{\otimes n}(\rho _{AR}^{\otimes n}) \, \big \Vert \, \mathcal {F}^{\otimes n}({\hat{\sigma }}_{A^nR^n}) \right)&\le \frac{1}{n} D_\alpha \! \left( \rho _{AR}^{\otimes n} \, \big \Vert \, {\hat{\sigma }}_{A^n R^n} \right) + \frac{1}{n} D^\text {reg}_\alpha \! \left( \mathcal {E}^{\otimes n} \, \big \Vert \, \mathcal {F}^{\otimes n} \right) \\&= \frac{1}{n} D_\alpha \! \left( \rho _{AR}^{\otimes n} \, \big \Vert \, {\hat{\sigma }}_{A^n R^n} \right) + D^\text {reg}_\alpha \! \left( \mathcal {E} \, \big \Vert \, \mathcal {F} \right) \,, \end{aligned}$$

where for the second line we used additivity of the regularised channel divergence (see Eq. (2.2)). Combining this with Eq. (3.14), we get

3.15 $$\begin{aligned} D_\alpha \! \left( \mathcal {E}(\rho _{AR}) \, \big \Vert \, \mathcal {F}(\sigma _{AR}) \right) \le \inf _{\hat{\sigma }_{A^n R^n} {\text { s.t. }}{\hat{\sigma }}_{A^n} = \sigma _A^{\otimes n}} \frac{1}{n} D_\alpha \! \left( \rho _{AR}^{\otimes n} \, \big \Vert \, {\hat{\sigma }}_{A^nR^n} \right) + D^\text {reg}_\alpha \! \left( \mathcal {E} \, \big \Vert \, \mathcal {F} \right) \,. \end{aligned}$$

Finally, using Lemma 3.3 and the fact that $d {:}{=}\dim (A)$ and $\alpha > 1$ are constants independent of *n*, we have

$$\begin{aligned}&\lim _{n \rightarrow \infty } \inf _{{\hat{\sigma }}_{A^n R^n} {\text { s.t. }}\hat{\sigma }_{A^n} = \sigma _A^{\otimes n}} \frac{1}{n} D_\alpha \! \left( \rho _{AR}^{\otimes n} \, \big \Vert \, {\hat{\sigma }}_{A^n R^n} \right) \\&\quad \le D_\alpha \! \left( \rho _A \, \big \Vert \, \sigma _A \right) + \lim _{n \rightarrow \infty } \frac{\alpha }{\alpha -1} \frac{d(d+1) \log (n+d)}{n} \\&\quad = D_\alpha \! \left( \rho _A \, \big \Vert \, \sigma _A \right) \,. \end{aligned}$$

Therefore, taking $n \rightarrow \infty$ in Eq. (3.15) and inserting this yields the theorem statement.$\quad \square$

### Removing the regularisation

The chain rule presented in Theorem 3.1 contains a regularised channel divergence term, which cannot be computed easily and whose behaviour as $\alpha \searrow 1$ is not understood. In this section we show that in the specific case relevant for entropy accumulation, this regularisation can be removed. From this, we then derive a chain rule for Rényi entropies in Theorem 3.6.

#### Definition 3.4

*(Replacer map).* The replacer map $\mathcal {S}_{A} \in \text {CP}(A,A)$ is defined by its action on an arbitrary state $\omega _{AR}$:

$$\begin{aligned} \mathcal {S}_A(\omega _{AR}) = {\mathbb {1}}_A \otimes \omega _{R} \,. \end{aligned}$$

Note that as usual, when we write $\mathcal {S}_A(\omega _{AR})$, we include an implicit tensoring with the identity channel, i.e. $\mathcal {S}_A(\omega _{AR}) = (\mathcal {S}_A \otimes \text {id}_R)(\omega _{AR})$.

#### Lemma 3.5

Let $\alpha \in (1,2)$, $\mathcal {E}\in \text {CPTP}(AR, A'R')$, and $\mathcal {F}= \mathcal {S}_{A'} \circ \mathcal {E}$, where $\mathcal {S}_{A'}$ is the replacer map. Then we have

$$\begin{aligned} D^\text {reg}_\alpha \! \left( \mathcal {E} \, \big \Vert \, \mathcal {F} \right) \le D_{\frac{1}{2 - \alpha }} \! \left( \mathcal {E} \, \big \Vert \, \mathcal {F} \right) \,. \end{aligned}$$

#### Proof

Due to the choice of $\mathcal {F}$, we have that for any state $\psi ^n \in \text {S}(A^nR^n {{\tilde{R}}}^n)$ (with ${{\tilde{R}}} \equiv AR$):

$$\begin{aligned} D_\alpha \! \left( \mathcal {E}^{\otimes n}(\psi ^n) \, \big \Vert \, \mathcal {F}^{\otimes n}(\psi ^n) \right) = - H_{\alpha }\left( (A')^{n} | (R')^{n} {{\tilde{R}}}^n \right) _{\mathcal {E}^{\otimes n}(\psi ^n)} \,. \end{aligned}$$

From [41, Proposition II.4] and [2, Lemma 4.2.2] we know that for every *n*, there exists a symmetric pure state $|{\hat{\psi }}^n\rangle \in \text {Sym}^n(A R {{\tilde{R}}})$ such that

$$\begin{aligned} D_\alpha \! \left( \mathcal {E}^{\otimes n} \, \big \Vert \, \mathcal {F}^{\otimes n} \right) = D_\alpha \! \left( \mathcal {E}^{\otimes n}({\hat{\psi }}^n) \, \big \Vert \, \mathcal {F}^{\otimes n}({\hat{\psi }}^n) \right) = - H_{\alpha }\left( (A')^{n} | (R')^{n} {{\tilde{R}}}^n \right) _{\mathcal {E}^{\otimes n}(\psi ^n)} \,, \end{aligned}$$

where ${\hat{\psi }}^n = |{\hat{\psi }}^n\rangle \!\langle {\hat{\psi }}^n|$ and the supremum in the definition of the channel divergence is achieved because the conditional entropy is continuous in the state. Let $d = \dim (AR{{\tilde{R}}})$ and $g_{n, d} = \dim (\text {Sym}^n(AR{{\tilde{R}}})) \le (n+1)^{d^2 - 1}$. We define the state

3.16 $$\begin{aligned} \tau ^n_{A^nR^n{{\tilde{R}}}^n} = \int \mu (\sigma _{AR{{\tilde{R}}}}) \sigma _{AR{{\tilde{R}}}}^{\otimes n} \,, \end{aligned}$$

where $\mu$ is the Haar measure on pure states. We now claim that in the limit $n\rightarrow \infty$, we can essentially replace the optimizer ${\hat{\psi }}^n_{A^nR^n{{\tilde{R}}}^n}$ by the state $\tau ^n_{A^nR^n{{\tilde{R}}}^n}$ in Eq. (3.16). More precisely, we claim that

3.17 $$\begin{aligned} \lim _{n \rightarrow \infty } \frac{1}{n} H_{\alpha }((A')^{n} | (R')^{n} \tilde{R}^n)_{\mathcal {E}^{\otimes n}({\hat{\psi }}^n)} \ge \lim _{n \rightarrow \infty } \frac{1}{n} H_{\frac{1}{2-\alpha }}((A')^{n} | (R')^{n} \tilde{R}^n)_{\mathcal {E}^{\otimes n}(\tau ^n)} \,. \end{aligned}$$

To show this, we first use Lemma 2.3 to getIt is know that $\tau ^n_{A^nR^n{{\tilde{R}}}^n}$ is the maximally mixed state on $\text {Sym}^n(AR{{\tilde{R}}})$ (see e.g. [13]). Therefore,

$$\begin{aligned} \rho ^n_{A^nR^n{{\tilde{R}}}^n} {:}{=}\frac{g_{n, d} \tau ^n - \hat{\psi }^n}{g_{n, d} - 1} \end{aligned}$$

is a valid quantum state (i.e. positive and normalised). Hence, we can write

$$\begin{aligned} \tau ^n = \left( 1 - \frac{1}{g_{n, d}} \right) \rho ^n + \frac{1}{g_{n, d}} {\hat{\psi }}^n \,. \end{aligned}$$

Using [1, Lemma B.5], it follows thatSince $\frac{\log (g_{n, d})}{n} \le (d^2 - 1) \frac{\log n}{n}$ vanishes as $n\rightarrow \infty$, taking the limit and using  proves Eq. (3.17).

Having established Eq. (3.17), we can now conclude the proof of the lemma as follows

$$\begin{aligned} D^\text {reg}_\alpha \! \left( \mathcal {E} \, \big \Vert \, \mathcal {F} \right)&= - \lim _{n \rightarrow \infty } \frac{1}{n} H_{\alpha }((A')^{n} | (R')^{n} \tilde{R}^n)_{\mathcal {E}^{\otimes n}({\hat{\psi }}^n)} \\&\le - \lim _{n \rightarrow \infty } \frac{1}{n} H_{\frac{1}{2-\alpha }}((A')^{n} | (R')^{n} \tilde{R}^n)_{\mathcal {E}^{\otimes n}(\tau ^n)} \\&= \lim _{n \rightarrow \infty } \frac{1}{n} D_{\frac{1}{2-\alpha }} \! \left( \mathcal {E}^{\otimes n}\Big ( \int \mu (\sigma _{AR{{\tilde{R}}}}) \sigma _{AR{{\tilde{R}}}}^{\otimes n} \Big ) \, \big \Vert \, \mathcal {F}^{\otimes n}\Big ( \int \mu (\sigma _{AR{{\tilde{R}}}}) \sigma _{AR{{\tilde{R}}}}^{\otimes n} \Big ) \right) \\&\le \lim _{n \rightarrow \infty } \sup _{\sigma _{AR{{\tilde{R}}}} \in \text {S}(AR{{\tilde{R}}})} \frac{1}{n} D_{\frac{1}{2-\alpha }} \! \left( \mathcal {E}^{\otimes n}\left( \sigma _{AR{{\tilde{R}}}}^{\otimes n} \right) \, \big \Vert \, \mathcal {F}^{\otimes n}\left( \sigma _{AR{{\tilde{R}}}}^{\otimes n} \right) \right) \\&= D_{\frac{1}{2-\alpha }} \! \left( \mathcal {E} \, \big \Vert \, \mathcal {F} \right) \,, \end{aligned}$$

where we used joint quasi-convexity [11, Proposition 4.17] in the fourth line and additivity under tensor products in the last line. $\square$

### Strengthened chain rule for conditional Rényi entropy

We next combine Theorem 3.1 with Lemma 3.5 to derive a new chain rule for the conditional Rényi entropy which then allows us to prove the generalised EAT in Sect. 4.

#### Lemma 3.6

Let $\alpha \in (1, 2)$, $\rho \in \text {S}(ARE)$, and $\mathcal {M}\in \text {CPTP}(RE, A'R'E')$ such that there exists $\mathcal {R}\in \text {CPTP}(E, E')$ such that $\text {Tr}_{A'R'} \circ \mathcal {M}= \mathcal {R}\circ \text {Tr}_{R}$. Then

3.18 $$\begin{aligned} H_{\alpha }(AA'|E')_{\mathcal {M}(\rho )} \ge H_{\alpha }(A|E)_{\rho } + \inf _{\omega \in \text {S}(RE{{\tilde{E}}})}H_{\frac{1}{2-\alpha }}(A'|E' \tilde{E})_{\mathcal {M}(\omega )} \end{aligned}$$

for a purifying system ${{\tilde{E}}} \equiv RE$.

#### Proof

We define the following maps^12^

$$\begin{aligned} \mathcal {N}&= \mathcal {S}_{A'} \circ \mathcal {M}  &   \in \text {CP}(RE, A'R'E')\,,\\ {\tilde{\mathcal {M}}}&= \text {id}_{A} \otimes \text {Tr}_{R'} \circ \mathcal {M}  &   \in \text {CPTP}(ARE, AA'E')\,,\\ {\tilde{\mathcal {N}}}&= \mathcal {S}_{A'} \circ {\tilde{\mathcal {M}}}  &   \in \text {CP}(ARE, AA'E') \,. \end{aligned}$$

Note that in Eq. (3.18), we can replace $\mathcal {M}$ by ${\tilde{\mathcal {M}}}$, as the system $R'$ does not appear in Eq. (3.18). With $\sigma _{ARE} = {\mathbb {1}}_A \otimes \rho _{RE}$ and ${\tilde{\mathcal {N}}} = \mathcal {S}_{A'} \circ {\tilde{\mathcal {M}}}$, we can write

$$\begin{aligned} - H_{\alpha }(AA'|E')_{\mathcal {M}(\rho )} = D_\alpha \! \left( {\tilde{\mathcal {M}}}(\rho _{ARE}) \, \big \Vert \, {\tilde{\mathcal {N}}}(\sigma _{ARE}) \right) \,. \end{aligned}$$

We now claim that there exists a map ${\tilde{\mathcal {R}}} \in \text {CP}(AE, AA'E)$ such that ${\tilde{\mathcal {N}}} = {\tilde{\mathcal {R}}} \circ \text {Tr}_{R}$. To see this, observe that by assumption, $\text {Tr}_{A'} \circ {\tilde{\mathcal {M}}} = \text {id}_{A} \otimes \mathcal {R}\circ \text {Tr}_{R}$ for some $\mathcal {R}\in \text {CP}(E, E')$. Then, we can define ${\tilde{\mathcal {R}}} \in \text {CP}(AE, AA'E)$ by its action on an arbitrary state $\omega _{AE}$:

$$\begin{aligned} {\tilde{\mathcal {R}}}(\omega _{AE}) {:}{=}{\mathbb {1}}_{A'} \otimes (\text {id}_A \otimes \mathcal {R})(\omega _{AE}) = {\mathbb {1}}_{A'} \otimes \text {Tr}_{A'} \circ {\tilde{\mathcal {M}}} (\omega _{ARE}) = {\tilde{\mathcal {N}}} (\omega _{ARE}) \end{aligned}$$

for any extension $\omega _{ARE}$ of $\omega _{AE}$. Therefore, we can apply Theorem 3.1 to find

$$\begin{aligned} D_\alpha \! \left( {\tilde{\mathcal {M}}}(\rho _{ARE}) \, \big \Vert \, {\tilde{\mathcal {N}}}(\sigma _{ARE}) \right) \le D_\alpha \! \left( \rho _{AE} \, \big \Vert \, \sigma _{AE} \right) + D^\text {reg}_\alpha \! \left( {\tilde{\mathcal {M}}} \, \big \Vert \, {\tilde{\mathcal {N}}} \right) \,. \end{aligned}$$

By definition of $\sigma$, we have $D_\alpha \! \left( \rho _{AE} \, \big \Vert \, \sigma _{AE} \right) = - H_{\alpha }(A|E)_{\rho }$. Since the channel divergence is stabilised (see Footnote 9), tensoring with $\text {id}_{A}$ has no effect, i.e.,

$$\begin{aligned} D^\text {reg}_\alpha \! \left( {\tilde{\mathcal {M}}} \, \big \Vert \, {\tilde{\mathcal {N}}} \right) = D^\text {reg}_\alpha \! \left( \text {Tr}_{R'} \circ \mathcal {M} \, \big \Vert \, \text {Tr}_{R'} \circ \mathcal {N} \right) = D^\text {reg}_\alpha \! \left( \text {Tr}_{R'} \circ \mathcal {M} \, \big \Vert \, \mathcal {S}_{A'} \circ \text {Tr}_{R'} \circ \mathcal {M} \right) \,. \end{aligned}$$

To this, we can apply Lemma 3.5 and obtain

$$\begin{aligned} D^\text {reg}_\alpha \! \left( {\tilde{\mathcal {M}}} \, \big \Vert \, {\tilde{\mathcal {N}}} \right) \le D_{\frac{1}{2 - \alpha }} \! \left( \text {Tr}_{R'} \circ \mathcal {M} \, \big \Vert \, \mathcal {S}_{A'} \circ \text {Tr}_{R'} \circ \mathcal {M} \right) = - \inf _{\omega \in \text {S}(RE{{\tilde{E}}})} H_{\frac{1}{2-\alpha }}(A' | E'{{\tilde{E}}})_{\mathcal {M}(\omega )} \end{aligned}$$

with ${{\tilde{E}}} \equiv RE$. Combining all the steps yields the desired statement. $\square$

## Generalised Entropy Accumulation

We are finally ready to state and prove the main result of this work which is a generalisation of the EAT proven in [1]. We first state a simple version of this theorem, which follows readily from the chain rule Theorem 3.6 and captures the essential feature of entropy accumulation: the min-entropy of a state $\mathcal {M}_n \circ \dots \circ \mathcal {M}_1(\rho )$ produced by applying a sequence of *n* channels can be lower-bounded by a sum of entropy contributions of each channel $\mathcal {M}_i$. However, for practical applications, it is desirable not to consider the state $\mathcal {M}_n \circ \dots \circ \mathcal {M}_1(\rho )$, but rather that state conditioned on some classical event, for example “success” in a key distribution protocol – a concept called “testing”. Analogously to [1], we present an EAT adapted to that setting in Sect. 4.2.

### Generalised EAT

#### Theorem 4.1

(Generalised EAT). Consider a sequence of channels $\mathcal {M}_i \in$ $\text {CPTP}(R_{i-1} E_{i-1}, A_i R_i E_i)$ such that for all $i \in \{1, \dots , n\}$, there exists $\mathcal {R}_i \in \text {CPTP}(E_{i-1}, E_i )$ such that $\text {Tr}_{A_i R_i} \circ \mathcal {M}_i = \mathcal {R}_i \circ \text {Tr}_{R_{i-1}}$. Then for any $\varepsilon \in (0,1)$ and any $\rho _{R_0 E_0 } \in \text {S}(R_0 E_0 )$

$$\begin{aligned} H_\text {min}^\varepsilon (A^n | E_n )_{\mathcal {M}_n \circ \dots \circ \mathcal {M}_1(\rho _{R_0 E_0 })} \ge \sum _{i = 1}^n \inf _{\omega \in \text {S}(R_{i-1}E_{i-1}{{\tilde{E}}}_{i-1})} H(A_i|E_i {{\tilde{E}}}_{i-1} )_{\mathcal {M}_i(\omega )} - O(\sqrt{n}) \end{aligned}$$

for a purifying system ${{\tilde{E}}}_{i-1} \equiv R_{i-1}E_{i-1}$. For a statement with explicit constants, see Eq. (4.1) in the proof.

#### Proof

By [1, Lemma B.10], we have for $\alpha \in (1,2)$

$$\begin{aligned} H_\text {min}^{\varepsilon }(A_1^n | E_n )_{\mathcal {M}_n \circ \dots \circ \mathcal {M}_1(\rho _{R_0 E_0 })} \ge H_{\alpha }(A_1^n | E_n )_{\mathcal {M}_n \circ \dots \circ \mathcal {M}_1(\rho _{R_0 E_0 })} - \frac{g(\varepsilon )}{\alpha -1} \end{aligned}$$

with $g(\varepsilon ) = \log (1 - \sqrt{1-\varepsilon ^2})$. From Theorem 3.6, we have

$$\begin{aligned}&H_{\alpha }(A_1^n | E_n )_{\mathcal {M}_n \circ \dots \circ \mathcal {M}_1(\rho _{R_0 E_0 })} \\&\hspace{20mm}\ge H_{\alpha }(A_1^{n-1} | E_{n-1} )_{\mathcal {M}_{n-1} \circ \dots \circ \mathcal {M}_1(\rho _{R_0 E_0 })} \\&\qquad + \inf _{\omega \in \text {S}(R_{n-1}E_{n-1}{{\tilde{E}}}_{n-1})} H_{\frac{1}{2-\alpha }}(A_n|E_n {{\tilde{E}}}_{n-1})_{\mathcal {M}_n(\omega )} \,. \end{aligned}$$

Repeating this step $n-1$ times, we get

$$\begin{aligned} H_{\alpha }(A_1^n | E_n )_{\mathcal {M}_n \circ \dots \circ \mathcal {M}_1(\rho _{R_0 E_0 })}&\ge H_{\alpha }(A_1 | E_1 )_{\mathcal {M}_1(\rho _{R_0 E_0 })} \\&\quad \ + \sum _{i = 2}^n \inf _{\omega \in \text {S}(R_{i-1}E_{i-1}{{\tilde{E}}}_{i-1})} H_{\frac{1}{2-\alpha }}(A_i|E_i {{\tilde{E}}}_{i-1})_{\mathcal {M}_i(\omega )} \\&\ge \sum _{i = 1}^n \inf _{\omega \in \text {S}(R_{i-1}E_{i-1}\tilde{E}_{i-1})} H_{\frac{1}{2-\alpha }}(A_i|E_i \tilde{E}_{i-1})_{\mathcal {M}_i(\omega )} \,, \end{aligned}$$

where the final step uses the monotonicity of the Rényi divergence in $\alpha$ [11, Corollary 4.3]. From [1, Lemma B.9] we have for each $i \in \{1, \dots , n\}$ and $\alpha$ sufficiently close to 1,

$$\begin{aligned}  &   \inf _{\omega \in \text {S}(R_{i-1}E_{i-1}{{\tilde{E}}}_{i-1})} H_{\frac{1}{2-\alpha }}(A_i|E_i {{\tilde{E}}}_{i-1})_{\mathcal {M}_i(\omega )}\\  &   \quad \ge \inf _{\omega \in \text {S}(R_{i-1}E_{i-1}{{\tilde{E}}}_{i-1})} H(A_i|E_i {{\tilde{E}}}_{i-1})_{\mathcal {M}_i(\omega )} - \frac{\alpha - 1}{2-\alpha } \log ^2\big (1 + 2 \, \dim (A_i)\big ) . \end{aligned}$$

Setting $d_A = \max _i \dim (A_i)$ and combining the previous steps, we obtain

4.1 $$\begin{aligned}  &   H_\text {min}(A_1^n | E_n )_{\mathcal {M}_n \circ \dots \circ \mathcal {M}_1(\rho _{R_0 E_0})} \nonumber \\  &   \ge \sum _{i = 1}^n \inf _{\omega _i \in \text {S}(R_{i-1}E_{i-1}\tilde{E}_{i-1})} H(A_i|E_i {{\tilde{E}}}_{i-1})_{\mathcal {M}_i(\omega _i)} - n \, \frac{\alpha - 1}{2-\alpha } \log ^2(1 + 2 d_A) - \frac{g(\varepsilon )}{\alpha -1} . \nonumber \\ \end{aligned}$$

Using $\alpha = 1 + O(1/\sqrt{n})$ yields the result. $\square$

### Generalised EAT with testing

In this section, we will extend Theorem 4.1 to include the possibility of “testing”, i.e., of computing the min-entropy of a cq-state conditioned on some classical event. This analysis is almost identical to that of [8]; we give the full proof for completeness, but will appeal to [8] for specific tight bounds. The resulting EAT (Theorem 4.3) has (almost) the same tight bounds as the result in [8], but replaces the Markov condition with the more general non-signalling condition. Hence, relaxing the Markov condition does not result in a significant loss in parameters (including second-order terms).

Consider a sequence of channels $\mathcal {M}_i \in \text {CPTP}(R_{i-1} E_{i-1}, C_i A_i R_i E_i)$ for $i \in \{1, \dots , n\}$, where $C_i$ are classical systems with common alphabet $\mathcal {C}$. We require that these channels $\mathcal {M}_i$ satisfy the following condition: defining $\mathcal {M}'_i = \text {Tr}_{C_i} \circ \mathcal {M}_i$, there exist channels $\mathcal {T}_i \in \text {CPTP}(A_i E_i, C_i A_i E_i)$ and $\mathcal {T}\in \text {CPTP}(A^n E_n, C^n A^n E_n)$ such that $\mathcal {M}_i = \mathcal {T}_i \circ \mathcal {M}'_i$ and $\mathcal {M}_n \circ \dots \circ \mathcal {M}_1 = \mathcal {T}\circ \mathcal {M}'_n \circ \dots \circ \mathcal {M}'_1$, where $\mathcal {T}_i$ and $\mathcal {T}$ have the form

4.2 $$\begin{aligned} \mathcal {T}_i(\omega _{A_i E_i}) = \sum _{y \in \mathcal {Y}_i , z \in \mathcal {Z}_i} (\Pi _{A_i}^{(y)} \otimes \Pi _{E_i}^{(z)}) \omega _{A_i E_i} (\Pi _{A_i}^{(y)} \otimes \Pi _{E_i}^{(z)}) \otimes |r_i(y,z)\rangle \!\langle r_i(y,z)|_{C_i} \,\nonumber \\ \mathcal {T}(\omega _{A^n E_n}) = \sum _{y \in \mathcal {Y}, z \in \mathcal {Z}} (\Pi _{A^n}^{(y)} \otimes \Pi _{E_n}^{(z)}) \omega _{A^n E_n} (\Pi _{A^n}^{(y)} \otimes \Pi _{E_n}^{(z)}) \otimes |r(y,z)\rangle \!\langle r(y,z)|_{C^n} \,, \end{aligned}$$

where $\{\Pi _{A_i}^{(y)}\}_y$ and $\{\Pi _{E_i}^{(z)}\}_z$ are families of mutually orthogonal projectors on $A_i$ and $E_i$, and $r_i: \mathcal {Y}_i \times \mathcal {Z}_i \rightarrow \mathcal {C}$ is a deterministic function Similarly, $\{\Pi _{A^n}^{(y)}\}_y$ and $\{\Pi _{E_n}^{(z)}\}_z$ are families of mutually orthogonal projectors on $A^n$ and $E_n$, and $r: \mathcal {Y}\times \mathcal {Z}\rightarrow \mathcal {C}$ is a deterministic function. (Note that even though we use the same symbol for both, in principle there does not have to be any relationship between the single-round projectors $\Pi _{A_i}$ and the projector $\Pi _{A^n}$ (and likewise for $\Pi _{E_i}$ and $\Pi _{E_n}$), although in practice the latter will usually be the tensor product of the former.) Intuitively, this condition says that for each round, the classical statistics can be reconstructed “in a projective way” from the systems $A_i$ and $E_i$ in that round, and furthermore the full statistics information $C^n$ can be reconstructed in a projective way from the systems $A^n$ and $E_n$ at the end of the process. The latter condition is not implied by the former because future rounds may modify the $E_i$-system in such a way that $C_i$ can no longer be reconstructed from the side information $E_n$ at the end of the protocol. To rule this out, we need to specify the latter condition separately. In particular, this requirement is always satisfied if the statistics $C_i$ are computed from classical information contained in $A_i$ and $E_i$ and this classical information is not deleted from $E_i$ in future rounds. This is the scenario in all applications that we are aware of, but we state Eq. (4.2) more generally to allow for the possibility of protocols where the statistics are constructed in a more general way.

Let $\mathbb {P}$ be the set of probability distributions on the alphabet $\mathcal {C}$ of $C_i$, and let ${{\tilde{E}}}_{i-1}$ be a system isomorphic to $R_{i-1} E_{i-1}$. For any $q \in \mathbb {P}$ we define the set of states

4.3 $$\begin{aligned} \Sigma _i(q) = \bigl \{\nu _{C_i A_i R_i E_i {{\tilde{E}}}_{i-1}} = \mathcal {M}_i(\omega _{R_{i-1} E_{i-1} {{\tilde{E}}}_{i-1}}) \,|\, \omega \in \text {S}(R_{i-1}E_{i-1}{{\tilde{E}}}_{i-1}) \text { and } \nu _{C_i} = q \bigr \} \ , \end{aligned}$$

where $\nu _{C_i}$ denotes the probability distribution over $\mathcal {C}$ with the probabilities given by $\textrm{Pr}\!\left[ c \right] = \langle c| \nu _{C_i} |c\rangle$. In other words, $\Sigma _i(q)$ is the set of states that can be produced at the output of the channel $\mathcal {M}_i$ and whose reduced state on $C_i$ is equal to the probability distribution *q*.

#### Definition 4.2

A function $f: \mathbb {P}\rightarrow \mathbb {R}$ is called a *min-tradeoff function* for $\{\mathcal {M}_i\}$ if it satisfies

$$\begin{aligned} f(q) \le \min _{\nu \in \Sigma _i(q)} H(A_i|E_i {{\tilde{E}}}_{i-1})_{\nu } \quad \forall i = 1, \dots , n\, . \end{aligned}$$

Note that if $\Sigma _i(q) = \emptyset$, then *f*(*q*) can be chosen arbitrarily.

Our result will depend on some simple properties of the tradeoff function, namely the maximum and minimum of *f*, the minimum of *f* over valid distributions, and the maximum variance of *f*:

$$\begin{aligned} \textsf{Max}(f)&{:}{=}\max _{q \in {\mathbb {P}}} f(q) \,,\\ \textsf{Min}(f)&{:}{=}\min _{q \in {\mathbb {P}}} f(q) \,,\\ \textsf{Min}_{\Sigma }(f)&{:}{=}\min _{q : \Sigma (q) \ne \emptyset } f(q) \,,\\ \textsf{Var}(f)&{:}{=}\max _{ q : \Sigma (q) \ne \emptyset } \sum _{x \in \mathcal {C}} q(x) f(\delta _{x})^2 - \left( \sum _{x \in \mathcal {C}} q(x) f(\delta _x) \right) ^2 \,, \end{aligned}$$

where $\Sigma (q) = \bigcup _i \Sigma _i(q)$ and $\delta _x$ is the distribution with all the weight on element *x*. We write $\textsf{freq}(C^n)$ for the distribution on $\mathcal {C}$ defined by $\textsf{freq}(C^n)(c) = \frac{|\{i \in \{1,\dots ,n\}: C_i = c\}|}{n}$. We also recall that in this context, an event $\Omega$ is defined by a subset of $\mathcal {C}^n$, and for a state $\rho _{C^n A^n E_n R_n}$ we write $\textrm{Pr}_{\rho }\!\left[ \Omega \right] = \sum _{c^n \in \Omega } \text{ Tr }\!\left[ \rho _{A_1^n E_n R_n, c^n} \right]$ for the probability of the event $\Omega$ and

$$\begin{aligned} \rho _{C^n A^n E_n R_n | \Omega } = \frac{1}{\textrm{Pr}_{\rho }\!\left[ \Omega \right] } \sum _{c^n \in \Omega } |c^n\rangle \!\langle c^n|_{C^n} \otimes \rho _{A^n E_n R_n, c^n} \end{aligned}$$

for the state conditioned on $\Omega$.

#### Theorem 4.3

Consider a sequence of channels $\mathcal {M}_i \in \text {CPTP}(R_{i-1}E_{i-1}, C_i A_i R_i E_i)$ for $i \in \{1, \dots , n\}$, where $C_i$ are classical systems with common alphabet $\mathcal {C}$ and the sequence $\{\mathcal {M}_i\}$ satisfies Eq. (4.2) and the non-signalling condition: for each $\mathcal {M}_i$, there exists $\mathcal {R}_i \in \text {CPTP}(E_{i-1}, E_i)$ such that $\text {Tr}_{A_i R_i C_i} \circ \mathcal {M}_i = \mathcal {R}_i \circ \text {Tr}_{R_{i-1}}$. Let $\varepsilon \in (0,1)$, $\alpha \in (1, 3/2)$, $\Omega \subset \mathcal {C}^n$, $\rho _{R_0 E_0} \in \text {S}(R_0 E_0)$, and *f* be an affine^13^ min-tradeoff function with $h = \min _{c^n \in \Omega } f(\textsf{freq}(c^n))$. Then,

4.4 $$\begin{aligned} H_\text {min}^\varepsilon (A^n | E_n)_{\mathcal {M}_n \circ \dots \circ \mathcal {M}_1(\rho _{R_0 E_0})_{|\Omega }}  &   \ge n \, h - n \, \frac{\alpha -1}{2-\alpha } \, \frac{\ln (2)}{2} V^2 - \frac{g(\varepsilon ) + \alpha \log (1/\textrm{Pr}_{\rho ^n}\!\left[ \Omega \right] )}{\alpha -1} \nonumber \\  &   \quad \ - n \, \left( \frac{\alpha -1}{2-\alpha } \right) ^2 K'(\alpha )\,, \end{aligned}$$

where $\textrm{Pr}\!\left[ \Omega \right]$ is the probability of observing event $\Omega$, and

$$\begin{aligned} g(\varepsilon )&= - \log (1 - \sqrt{1-\varepsilon ^2}) \,,\\ V&= \log (2d_A^2+1) + \sqrt{2 + \textsf{Var}(f)} \,,\\ K'(\alpha )&= \frac{(2-\alpha )^3}{6 (3-2\,\alpha )^3 \ln 2} \, 2^{\frac{\alpha -1}{2-\alpha }(2\log d_{A} + \textsf{Max}(f) - \textsf{Min}_{\Sigma }(f))} \ln ^3\left( 2^{2\log d_{A} + \textsf{Max}(f) - \textsf{Min}_{\Sigma }(f)} + e^2 \right) \,, \end{aligned}$$

with $$d_A = \max _i \dim (A_i)$$.

#### Remark 4.4

The parameter in $\alpha$ in Theorem 4.3 can be optimized for specific problems, which leads to tighter bounds. Alternatively, it is possible to make a generic choice for $\alpha$ to recover a theorem that looks much more like Theorem 4.1, which is done in Corollary 4.6. We also remark that even tighter second order terms have been derived in [42]. To keep our theorem statement and proofs simpler, we do not carry out this additional optimization explicitly, but note that this can be done in complete analogy to [42].

To prove Theorem 4.3, we will need the following lemma (which is already implicit in [1, Claim 4.6], but we give a simplified proof here).

#### Lemma 4.5

Consider a quantum state $\rho \in \text {S}(CADE)$ that has the form

$$\begin{aligned} \rho _{CADE} = \sum _{c \in \Omega } |c\rangle \!\langle c| \otimes \rho _{AE,c} \otimes \rho _{D|c} \,, \end{aligned}$$

where $\Omega \subset \mathcal {C}$ is a subset of the alphabet $\mathcal {C}$ of the classical system *C*, and for each *c*, $\rho _{AE,c} \in {{\,\textrm{Pos}\,}}(AE)$ is subnormalised and $\rho _{D|c} \in \text {S}(D)$ is a quantum state. Then for $\alpha > 1$,

#### Proof

Let $\sigma _E \in \text {S}(E)$ such thatThen

$$\begin{aligned} \left( \sigma _E^{\frac{1-\alpha }{2 \alpha }} \rho _{CADE} \sigma _E^{\frac{1-\alpha }{2 \alpha }} \right) ^{\alpha } = \sum _{c \in \Omega } |c\rangle \!\langle c| \otimes \left( \sigma _E^{\frac{1-\alpha }{2 \alpha }} \rho _{AE,c} \sigma _E^{\frac{1-\alpha }{2 \alpha }} \right) ^{\alpha } \otimes \rho _{D|c}^{\alpha } \,. \end{aligned}$$

Hence,

$$\begin{aligned} \text{ Tr }\!\left[ \left( \sigma _E^{\frac{1-\alpha }{2 \alpha }} \rho _{CADE} \sigma _E^{\frac{1-\alpha }{2 \alpha }} \right) ^{\alpha } \right]&= \sum _{c \in \Omega } \text{ Tr }\!\left[ \left( \sigma _E^{\frac{1-\alpha }{2 \alpha }} \rho _{AE,c} \sigma _E^{\frac{1-\alpha }{2 \alpha }} \right) ^{\alpha } \right] \, \text{ Tr }\!\left[ \rho _{D|c}^{\alpha } \right] \\&\le \sup _{{\tilde{\sigma }}_E \in \text {S}(E)} \text{ Tr }\!\left[ \sum _{c \in \Omega } |c\rangle \!\langle c| \otimes \left( {\tilde{\sigma }}_E^{\frac{1-\alpha }{2 \alpha }} \rho _{AE,c} {\tilde{\sigma }}_E^{\frac{1-\alpha }{2 \alpha }} \right) ^{\alpha } \right] \\  &\quad \ \times \max _{c \in \Omega } \text{ Tr }\!\left[ \rho _{D|c}^{\alpha } \right] \\&= \sup _{{\tilde{\sigma }}_E \in \text {S}(E)} \text{ Tr }\!\left[ \left( \tilde{\sigma }_E^{\frac{1-\alpha }{2 \alpha }} \rho _{CAE} \tilde{\sigma }_E^{\frac{1-\alpha }{2 \alpha }} \right) ^{\alpha } \right] \, \max _{c \in \Omega } \text{ Tr }\!\left[ \rho _{D|c}^{\alpha } \right] \end{aligned}$$

Recalling the definitions of $D_{\alpha }$ (Definition 2.1) and  (Definition 2.2), we see that the lemma follows by taking the logarithm and multiplying by $\frac{1}{\alpha - 1}$. $\square$

#### Proof of Theorem 4.3

As in the proof of Theorem 4.1, we first use [1, Lemma B.10] to get4.5for $\alpha \in (1,2]$ and $g(\varepsilon ) = \log (1 - \sqrt{1-\varepsilon ^2})$. We therefore need to find a lower bound for4.6where the equality holds because of Eq. (4.2) and [1, Lemma B.7].

Before proceeding with the formal proof, let us explain the main difficulty compared to Theorem 4.1. The state for which we need to compute the entropy in Eq. (4.6) is conditioned on the event $\Omega \subset \mathcal {C}^n$. This is a global event, in the sense that it depends on the classical outputs $C_1, \dots , C_n$ of all rounds. We essentially seek a lower bound that involves $\min _{\nu \in \Sigma _i(\textsf{freq}(c^n))} H_{\alpha }(A_i|E_i )_{\nu }$ for some $c^n \in \Omega$, i.e., for every round we only want to minimize over output states of the channel $\mathcal {M}_i$ whose distribution on $C_i$ matches the frequency distribution $\textsf{freq}(c^n)$ of the *n* rounds we observed. This means that we must use the global conditioning on $\Omega$ to argue that in each round, we can restrict our attention to states whose outcome distribution matches the (worst-case) frequency distribution associated with $\Omega$. The chain rule Theorem 3.1 does not directly allow us to do this as the r.h.s. of Eq. (3.18) always minimizes over all possible input states.

To circumvent this, we follow a strategy that was introduced in [1] and optimized in [8] (see also [16, 21, 43] for related ideas and [44] for follow-up work). For every *i*, we introduce a quantum system $D_i$ with $\dim (D_i) = \lceil 2^{\textsf{Max}(f) - \textsf{Min}(f)} \rceil$ and define $\mathcal {D}_i \in \text {CPTP}(C_i, C_i D_i)$ by

$$\begin{aligned} \mathcal {D}_i(\omega _{C_i}) = \sum _{c \in \mathcal {C}} \langle c|\omega _{C_i}|c\rangle \cdot |c\rangle \!\langle c| \otimes \tau _{D_{i}|c} \,. \end{aligned}$$

For every $c \in \mathcal {C}$, the state $\tau _{D_{i}|c} \in \text {S}(D)$ is defined as the mixture between a uniform distribution on $\{1, \dots , \lfloor 2^{\textsf{Max}(f) - f(\delta _c)} \rfloor \}$ and a uniform distribution on $\{1, \dots , \lceil 2^{\textsf{Max}(f) - f(\delta _c)} \rceil \}$ that satisfies

$$\begin{aligned} H(D_i)_{\tau _{D_i|c}} = \textsf{Max}(f) - f(\delta _c) \, , \end{aligned}$$

where $\delta _x$ stands for the distribution with all the weight on element *x*. This is clearly possible if $\dim (D_i) = \lceil 2^{\textsf{Max}(f) - \textsf{Min}(f)} \rceil$.

We define ${\bar{\mathcal {M}}}_i = \mathcal {D}_i \circ \mathcal {M}_i$ and denote

$$\begin{aligned} \rho ^n_{C^n A^n R_n E_n } = \mathcal {M}_n \circ \dots \circ \mathcal {M}_1(\rho _{R_0 E_0 }) \;\;\text { and }\;\; {\bar{\rho }}^n_{C^n A^n D^n R_n E_n } = {\bar{\mathcal {M}}}_n \circ \dots \circ {\bar{\mathcal {M}}}_1(\rho _{R_0 E_0 }) \,. \end{aligned}$$

The state ${\bar{\rho }}^n_{|\Omega }$ has the right form for us to apply Lemma 4.5 and get4.7where

$$\begin{aligned} {\bar{\rho }}^n_{D^n|c^n} = \tau _{D_{1}|c_1} \otimes \dots \otimes \tau _{D_{n}|c_n} \,. \end{aligned}$$

We treat each term in Eq. (4.7) in turn.

(i) For the term on the l.h.s., it is easy to see that $\bar{\rho }^n_{C^n A^n R_n E_n |\Omega } = \rho ^n_{C^n A^n R_n E_n |\Omega }$, so 4.8
(ii) For the first term on the r.h.s., we compute
  4.9 $$\begin{aligned} H_{\alpha }(D^n)_{{\bar{\rho }}^n_{D^n|c^n}} = \sum _{i} H_{\alpha }(D_i)_{\tau _{D_{i}|c_i}} \le \sum _{i} H(D_i)_{\tau _{D_{i}|c_i}}&= n\,\textsf{Max}(f) - \sum _{i} f(\delta _{c_i}) \nonumber \\&= n\,\textsf{Max}(f) - n f(\textsf{freq}(c^n)) \,, \end{aligned}$$
  where the last equality holds because *f* is affine.
(iii) For the second term on the r.h.s., we first use [1, Lemma B.5] to remove the conditioning on the event $\Omega$, and then use that removing the classical system $C^n$ and switching from  to $H_{\alpha }$ can only decrease the entropy:  where we used $\textrm{Pr}_{\rho ^n}\!\left[ \Omega \right] = \textrm{Pr}_{{\bar{\rho }}^n}\!\left[ \Omega \right]$. Now noting that $\text {Tr}_{D_i} \circ {\bar{\mathcal {M}}}_i = \mathcal {M}_i$, we see that the non-signalling condition $\text {Tr}_{A_i R_i C_i} \circ \mathcal {M}_i = \mathcal {R}_i \circ \text {Tr}_{R_{i-1}}$ on $\mathcal {M}_i$ implies the non-signalling condition $\text {Tr}_{A_i R_i C_i D_i} \circ \bar{\mathcal {M}}_i = \mathcal {R}_i \circ \text {Tr}_{R_{i-1}}$ on ${\bar{\mathcal {M}}}_i$. We can therefore apply the chain rule in Theorem 3.6 to find
  $$\begin{aligned} H_{\alpha }(A^n D^n|E_n )_{{\bar{\rho }}^n} \ge \sum _{i = 1}^n \min _{\omega _{i-1} \in \text {S}(R_{i-1} E_{i-1} {{\tilde{E}}}_{i-1})} H_{\beta }(A_i D_i|E_i {{\tilde{E}}}_{i-1})_{\bar{\mathcal {M}}_i(\omega _{i-1})} \,, \end{aligned}$$
  where we introduced the shorthand $\beta {:}{=}\frac{1}{2-\alpha }$ and the purifying system ${{\tilde{E}}}_{i-1} \equiv R_{i-1} E_{i-1}$. Noting that for $\alpha \in (1,3/2)$ we have $\beta \in (1, 2)$, we can now use [8, Corollary IV.2] to obtain
  $$\begin{aligned}&H_{\beta }(A_i D_i|E_i {{\tilde{E}}}_{i-1})_{\bar{\mathcal {M}}_i(\omega _{i-1})} \\&\quad \ge H(A_i D_i|E_i {{\tilde{E}}}_{i-1})_{\bar{\mathcal {M}}_i(\omega _{i-1})} - (\beta - 1)\frac{\ln (2)}{2} V^2 - (\beta -1)^2 K( \beta ) \,, \end{aligned}$$
  where $V^2$ and $K(\beta )$ are quantities from [8, Proposition V.3] that satisfy
  $$\begin{aligned} K(\beta )&\le \frac{1}{6 (2-\beta )^3 \ln 2} \,\\&2^{(\beta - 1)(2\log d_{A} + \textsf{Max}(f) - \textsf{Min}_{\Sigma }(f))} \ln ^3\left( 2^{2\log d_{A} + \textsf{Max}(f) - \textsf{Min}_{\Sigma }(f)} + e^2 \right) \,, \\ V^2&= \left( \log (2d_A^2+1) + \sqrt{2 + \textsf{Var}(f)} \right) ^2 \,, \end{aligned}$$
  where $d_A = \max _i \dim (A_i)$. Note that the above expressions derived in [8, Proposition V.3] also hold in our case due to the first part of Eq. (4.2). Furthermore, as in the proof of [8, Proposition V.3], we have
  $$\begin{aligned} H(A_i D_i|E_i {{\tilde{E}}}_{i-1})_{{\bar{\mathcal {M}}}_i(\omega _{i-1})} \ge \textsf{Max}(f) \,. \end{aligned}$$
  Therefore, the second term on the r.h.s. of Eq. (4.7) is bounded by 4.10

Combining our results for each of the three terms (i.e. Eqs. (4.8), (4.9) and (4.10)) and recalling $h = \min _{x^n \in \Omega } f(\textsf{freq}(x^n))$, Eq. (4.7) becomesInserting this into Eqs. (4.5) and (4.6), and defining $K'(\alpha ) = K(\beta ) = K(\frac{1}{2-\alpha })$ we obtain

4.11 $$\begin{aligned} H_\text {min}^\varepsilon (A^n | E_n )_{\mathcal {M}_n \circ \dots \circ \mathcal {M}_1(\rho _{R_0 E_0 })_{|\Omega }}  &   \ge n \, h - n \, (\beta - 1)\frac{\ln (2)}{2} V^2 - \frac{g(\varepsilon ) + \alpha \log (1/\textrm{Pr}_{\rho ^n}\!\left[ \Omega \right] )}{\alpha -1}\nonumber \\  &   \quad \ - n \, (\beta - 1)^2 K(\beta ) \end{aligned}$$

as desired. $$\square $$

#### Corollary 4.6

For the setting given in Theorem 4.3 we have

$$\begin{aligned} H_\text {min}^\varepsilon (A^n | E_n )_{\mathcal {M}_n \circ \dots \circ \mathcal {M}_1(\rho _{R_0 E_0 })_{|\Omega }} \ge n h - c_1 \sqrt{n} - c_0 \, , \end{aligned}$$

where the quantities $c_1$ and $c_0$ are given by

$$\begin{aligned} c_1&= \sqrt{ \frac{2 \ln (2) V^2}{\eta }\Big (g(\varepsilon ) + (2 - \eta ) \log (1/\textrm{Pr}_{\rho ^n}\!\left[ \Omega \right] ) \Big )}\,,\\ c_0&= \frac{(2 - \eta ) \eta ^2 \log (1/\textrm{Pr}_{\rho ^n}\!\left[ \Omega \right] ) + \eta ^2 g(\varepsilon )}{3 (\ln 2)^2 V^2 (2\eta -1)^3} \,\\&\qquad 2^{\frac{1-\eta }{\eta }(2\log d_{A} + \textsf{Max}(f) - \textsf{Min}_{\Sigma }(f))} \ln ^3\left( 2^{2\log d_{A} + \textsf{Max}(f) - \textsf{Min}_{\Sigma }(f)} + e^2 \right) \end{aligned}$$

with

$$\begin{aligned} \eta&= \frac{2 \ln (2)}{1 + 2 \ln (2)} \,, \qquad g(\varepsilon ) = \log (1 - \sqrt{1-\varepsilon ^2}) \,, \qquad V = \log (2d_A^2+1) + \sqrt{2 + \textsf{Var}(f)} \,. \end{aligned}$$

#### Proof

We first note that for any $\Omega$ with non-zero probability, $h \le \log d_A$. Therefore, if $n \le \left( \frac{c_1}{2 \log d_A} \right) ^2$, it is easy to check that $n h - c_1 \sqrt{n} \le - n \log d_A$, so the statement of Corollary 4.6 becomes trivial. We may therefore assume that $n \ge ( \frac{c_1}{2 \log d_A})^2$.

As in the proof of Theorem 4.3, we define $\beta = \frac{1}{2-\alpha }$. The first part of the proof works for any $\alpha \in (1, 2-\eta )$ for $\eta = \frac{2 \ln (2)}{1+ 2 \ln (2)} \approx 0.58$; later we will make a specific choice of $\alpha$ in this interval. Then, $\beta - 1 = \frac{1}{2 - \alpha } - 1 \le \frac{\alpha - 1}{\eta }$ and $\beta \in (1, 1/\eta )$. Therefore, using $K(\beta )$ as defined in the proof of Theorem 4.3 and noting that in the interval $\beta \in (1, 1/\eta ) \subset (1,2)$ this quantity is monotonically increasing in $\beta$, we have

$$\begin{aligned}&K(\beta ) \le K {:}{=}\\&\frac{\eta ^3}{6 (2 \eta - 1)^3 \ln 2} \, 2^{\frac{1-\eta }{\eta }(2\log d_{A} + \textsf{Max}(f) - \textsf{Min}_{\Sigma }(f))} \ln ^3\left( 2^{2\log d_{A} + \textsf{Max}(f) - \textsf{Min}_{\Sigma }(f)} + e^2 \right) \,, \end{aligned}$$

Hence, we can simplify the statement of Theorem 4.3 to

4.12 $$\begin{aligned}  &   H_\text {min}^\varepsilon (A^n | E_n )_{\mathcal {M}_n \circ \dots \circ \mathcal {M}_1(\rho _{R_0 E_0 })_{|\Omega }} \nonumber \\  &   \ge n \, h - n \, (\alpha - 1) \frac{\ln (2)}{2 \eta } V^2 - \frac{g(\varepsilon ) + (2-\eta ) \cdot \log (1/\textrm{Pr}_{\rho ^n}\!\left[ \Omega \right] )}{\alpha -1} - n \, (\alpha - 1)^2 \frac{K}{\eta ^2}. \nonumber \\ \end{aligned}$$

We now choose $\alpha > 1$ as a function of *n* and $\varepsilon$ so that the terms proportional to $\alpha - 1$ and $\frac{1}{\alpha -1}$ match:

$$\begin{aligned} \alpha = 1+ \sqrt{\frac{2 \eta }{n \ln (2) V^2 }\Big ( g(\varepsilon ) + (2 - \eta ) \log (1/\textrm{Pr}_{\rho ^n}\!\left[ \Omega \right] ) \Big )} \,. \end{aligned}$$

Inserting this choice of $\alpha$ into Eq. (4.12) and combining terms yields the constants in Corollary 4.6. The final step is to show that this choice of $\alpha$ indeed satisfies $\alpha \le 2-\eta$ for $n \ge ( \frac{c_1}{2 \log d_A})^2$. For this, we note that for $n \ge ( \frac{c_1}{2 \log d_A})^2$, we have

$$\begin{aligned} \alpha = 1 + \frac{\eta }{\ln (2) V^2} \frac{c_1}{\sqrt{n}} \le 1 + \frac{2 \eta \log d_A}{\ln (2) V^2} \,. \end{aligned}$$

We can now use that $V^2 \ge \left( \log (2 d_A^2) \right) ^2 \ge 4 \log d_A$ since $d_A \ge 2$, so

$$\begin{aligned} \alpha \le 1 + \frac{2 \eta \log d_A}{\ln (2) V^2} \le 1 + \frac{\eta }{2 \ln (2)} = 2 - \eta \,, \end{aligned}$$

where the last inequality holds because $\eta = \frac{2 \ln (2)}{1 + 2 \ln (2)}$. $\square$

In many applications, e.g. randomness expansion or QKD, a round can either be a “data generation round” (e.g. to generate bits of randomness or key) or a “test round” (e.g. to test whether a device used in the protocol behaves as intended). More formally, in this case the maps $\mathcal {M}_i \in \text {CPTP}(R_{i-1}E_{i-1}, C_i A_i R_i E_i)$ can be written as

4.13 $$\begin{aligned} \mathcal {M}_i = \gamma \mathcal {M}^{\text {test}}_{i, R_{i-1}E_{i-1} \rightarrow C_i A_i R_i E_i} + (1-\gamma ) \mathcal {M}^{\text {data}}_{i, R_{i-1}E_{i-1} \rightarrow A_i R_i E_i} \otimes |\bot \rangle \!\langle \bot |_{C_i} \,, \end{aligned}$$

where the output of $\mathcal {M}^{\text {test}}_i$ on system $C_i$ is from some alphabet $\mathcal {C}'$ that does not include $\bot$, so the alphabet of system $C_i$ is $\mathcal {C}= \mathcal {C}' \cup \{\bot \}$. The parameter $\gamma$ is called the *testing probability*, and for efficient protocols we usually want $\gamma$ to be as small as possible.

For maps of the form in Eq. (4.13), there is a general way of constructing a min-tradeoff function for the map $\mathcal {M}_i$ based only on the statistics generated by the map $\mathcal {M}^{\text {test}}_i$. This was shown in [8] and we reproduce their result (adapted to our notation) here for the reader’s convenience.

#### Lemma 4.7

([8, Lemma V.5]). Let $\mathcal {M}_i \in \text {CPTP}(R_{i-1}E_{i-1}, C_i A_i R_i E_i)$ be channels satisfying the same conditions as in Theorem 4.3 that can furthermore be decomposed as in Eq. (4.13). Suppose that an affine function $g: \mathbb {P}(\mathcal {C}') \rightarrow \mathbb {R}$ satisfies for any $q' \in \mathbb {P}(\mathcal {C}')$ and any $i = 1, \dots , n$

4.14 $$\begin{aligned} g(q') \le \min _{\omega \in \text {S}(R_{i-1} E_{i-i} {{\tilde{E}}}_{i-1})} \bigl \{ H(A_i | E_i {{\tilde{E}}}_{i-1})_{\mathcal {M}_i(\omega )}: \left( \mathcal {M}^{\text {test}}_i(\omega )\right) _{C_i} = q' \bigr \} \end{aligned}$$

where ${{\tilde{E}}}_{i-1} \equiv R_{i-1} E_{i-1}$ is a purifying system. Then, the affine function $f: \mathbb {P}(\mathcal {C}) \rightarrow \mathbb {R}$ defined by

$$\begin{aligned} f(\delta _x)&= \textsf{Max}(g) + \frac{1}{\gamma } (g(\delta _x) - \textsf{Max}(g)) \;\;\;\;\; \forall x \in \mathcal {C}'\\ f(\delta _{\bot })&= \textsf{Max}(g) \end{aligned}$$

is a min-tradeoff function for $\{\mathcal {M}_{i}\}$. Moreover,

$$\begin{aligned} \textsf{Max}(f)&= \textsf{Max}(g) \\ \textsf{Min}(f)&= \left( 1-\frac{1}{\gamma } \right) \textsf{Max}(g) + \frac{1}{\gamma }\textsf{Min}(g) \\ \textsf{Min}_{\Sigma }(f)&\ge \textsf{Min}(g) \\ \textsf{Var}(f)&\le \frac{1}{\gamma }\big (\textsf{Max}(g) - \textsf{Min}(g) \big )^2. \end{aligned}$$

## Sample Applications

To demonstrate the utility of our generalised EAT, we provide two sample applications. Firstly, in Sect. 5.1 we prove security of blind randomness expansion against general attacks. The notion of blind randomness was defined in [15] and has potential applications in mistrustful cryptography (see [15, 16] for a detailed motivation). Until now, no security proof against general attacks was known. In particular, the original EAT is not applicable because its model of side information is too restrictive. With our generalised EAT, we can show that security against general attacks follows straightforwardly from a single-round security statement.

Secondly, in Sect. 5.2 we give a simplified security proof for the E91 QKD protocol [45], which was also treated with the original EAT [1]. This example is meant to help those familiar with the original EAT understand the difference between that result and our generalised EAT. In particular, this application highlights the utility of our more general model of side information: in our proof, the non-signalling condition is satisfied trivially and the advantage over the original EAT stems purely from being able to update the side information register $E_i$. We point out that while here we focus on the E91 protocol to allow an easy comparison with the original EAT, our generalised EAT can be used for a large class of QKD protocols for which the original EAT was not applicable at all. A comprehensive treatment of this is given in [7].

### Blind randomness expansion

We start by recalling the idea of standard (non-blind) device-independent randomness expansion [17–21]. Alice would like to generate a uniformly random bit string using devices $D_1$ and $D_2$ prepared by an adversary Eve. To this end, in her local lab (which Eve cannot access) she isolates the devices from one another and plays multiple round of a non-local game with them, e.g. the CHSH game. On a subset of the rounds of the game, she checks whether the CHSH condition is satisfied. If this is the case on a sufficiently high proportion of rounds, she can conclude that the devices’ outputs on the remaining rounds must contain a certain amount of entropy, conditioned on the input to the devices and any quantum side information that Eve might have kept from preparing the devices. Using a quantum-proof randomness extractor, Alice can then produce a uniformly random string.

Blind randomness expansion [15, 16] is a significant strengthening of the above idea. Here, Alice only receives one device $D_1$, which she again places in her local lab isolated from the outside world. Now, Alice plays a non-local game with her device $D_1$ and the adversary Eve: she samples questions for a non-local game as before, inputs one of the questions to $D_1$, and sends the other question to Eve. $D_1$ and Eve both provide an output. Alice then proceeds as in standard randomness expansion, checking whether the winning condition of the non-local game is satisfied on a subset of rounds and concluding that the output of her device $D_1$ must contain a certain amount of entropy conditioned on the adversary’s side information.

For the purpose of applying the EAT, the crucial difference between the two notions of randomness expansion is the following: in standard randomness expansion, the adversary’s quantum side information is not acted upon during the protocol, and additional side information (the inputs to the devices, which we also condition on) are generated independently in a round-by-round manner. This allows a relatively straightforward application of the standard EAT [4]. In contrast, in blind randomness expansion, the adversary’s quantum side information gets updated in every round of the protocol and is not generated independently in a round-by-round fashion. This does not fit in the framework of the standard EAT, which requires the side information to be generated round-by-round subject to a Markov condition. As a result, [15, 16] were not able to prove a general multi-round blind randomness expansion result.

In the rest of this section, we will show that our generalised EAT is capable of treating multi-round blind randomness expansion, using a protocol similar to [14, Protocol 3.1]. A formal description of the protocol is given in Protocol 1.
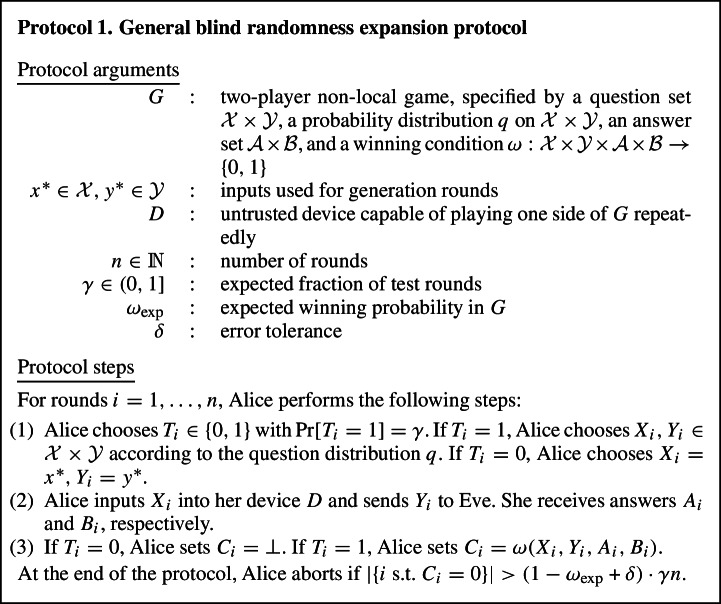


The following proposition shows a lower bound on on the amount of randomness Alice can extract from this protocol, as specified by the min-entropy. For this, we assume a lower-bound on the single-round von Neumann entropy. Such a single-round bound can be found numerically using a generic method as explained after the proof of Lemma 5.1.

#### Proposition 5.1

Suppose Alice executes Protocol 1 with a device *D* that cannot communicate with Eve. We denote by $R_i$ and $E'_i$ the (arbitrary) quantum systems of the device *D* and the adversary Eve after the *i*-th round, respectively. Eve’s full side-information after the *i*-th round is $E_i {:}{=}T^i X^i Y^i B^i E'_i$. A single round of the protocol can be described by a quantum channel $\mathcal {N}_i \in \text {CPTP}(R_{i-1} E_{i-1}, C_i A_i R_i E_i)$. We also define $\mathcal {N}_i^{\text {test}}$ to be the same as $\mathcal {N}_i$, except that $\mathcal {N}_i^{\text {test}}$ always picks $T_i = 1$. Let $\rho _{A^n C^n R_n E_n}$ be the state at the end of the protocol and $\Omega$ the event that Alice does not abort.

Let $g: \mathbb {P}(\{0, 1\}) \rightarrow \mathbb {R}$ be an affine function satisfying the conditions

5.1 $$\begin{aligned} g(p) \le \inf _{\omega \in \text {S}(R_{i-1}E_{i-1}{{\tilde{E}}}_{i-1}) : \, \mathcal {N}_i^{\text {test}}(\omega )_{C_i} = p} H(A_i|E_i \tilde{E}_{i-1})_{\mathcal {N}_i(\omega )} \,, \qquad \textsf{Max}(g) = g(\delta _1) \,, \end{aligned}$$

where ${{\tilde{E}}}_{i-1} \equiv R_{i-1}E_{i-1}$ is a purifying system. Then, for any $\varepsilon _a, \varepsilon _s \in (0,1)$, either $\textrm{Pr}\!\left[ \Omega \right] \le \varepsilon _a$ or

$$\begin{aligned} H_\text {min}^{\varepsilon _s} (A^n | E_n)_{\rho _{|\Omega }} \ge n h - c_1 \sqrt{n} - c_0 \end{aligned}$$

for $c_1, c_0 \ge 0$ independent of *n* and

$$\begin{aligned} h&= \min _{p' \in \mathbb {P}(\{0, 1\}): p'(0) \le 1 - \omega _{\text {exp}}+ \delta } g(p') \,, \end{aligned}$$

where $\omega _{\text {exp}}$ is the expected winning probability and $\delta$ the error tolerance from Protocol 1. If we treat $\varepsilon _s, \varepsilon _a, \dim (A_i), \delta , \textsf{Max}(g),$ and $\textsf{Min}(g)$ as constants, then $c_1 = O(1/\sqrt{\gamma })$ and $c_0 = O(1)$.

Furthermore, if there exists a quantum strategy that wins the game *G* with probability $\omega _{\text {exp}}$, there is an honest behaviour of *D* and Eve for which $\textrm{Pr}\!\left[ \Omega \right] \ge 1 - \exp (-\frac{\delta ^2}{1 - \omega _{\text {exp}}+ \delta } \gamma n)$.

#### Remark 5.2

The condition on *g*(*p*) in Eq. (5.1) is formulated in terms of the entropy

$$\begin{aligned} H(A_i|E_i {{\tilde{E}}}_{i-1})_{\mathcal {N}_i(\omega )} = H(A_i|T^i X^i Y^i B^i E'_i {{\tilde{E}}}_{i-1})_{\mathcal {N}_i(\omega )} \end{aligned}$$

with ${{\tilde{E}}}_{i-1} \equiv R_{i-1} E_{i-1}$. However, the map $\mathcal {N}_i$ corresponding to the *i*-th round does not act on the systems $T^{i-1} X^{i-1} Y^{i-1} B^{i-1}$. Therefore, we can view these systems as part of the purifying system. Since the infimum in Eq. (5.1) already includes a purifying ${{\tilde{E}}}_{i-1}$, we can drop these additional systems and without loss of generality choose ${{\tilde{E}}}_{i-1}$ to be isomorphic to those input systems on which $\mathcal {N}_i$ acts non-trivially, i.e. ${{\tilde{E}}}_{i-1} \equiv R_{i-1} E'_{i-1}$. This means that we can replace the upper bound on *g* in Eq. (5.1) by the equivalent condition

5.2 $$\begin{aligned} g(p) \le \inf _{\omega \in \text {S}(R_{i-1}E_{i-1}{{\tilde{E}}}_{i-1}) : \, \mathcal {N}_i^{\text {test}}(\omega )_{C_i} = p} H(A_i|B_i X_i Y_i T_i E'_i {{\tilde{E}}}_{i-1})_{\mathcal {N}_i(\omega )} \end{aligned}$$

with ${{\tilde{E}}}_{i-1} \equiv R_{i-1} E'_{i-1}$. For the proof of Lemma 5.1 we will use Eq. (5.1) since it more closely matches the notation of Theorem 4.3, but intuitively, Eq. (5.2) is more natural as it only involves quantities related to the *i*-th round of the protocol.

#### Proof of Lemma 5.1

To show the min-entropy lower bound, we will make use of Corollary 4.6. For this, we first check that the maps $\mathcal {N}_i$ satisfy the required conditions. Since $C_i$ is a deterministic function of the (classical) variables $X_i, Y_i, A_i,$ and $B_i$, it is clear that Eq. (4.2) is satisfied. For the non-signalling condition, we define the map $\mathcal {R}_i \in \text {CPTP}(E_{i-1}, E_i)$ as follows: $\mathcal {R}_i$ samples $T_i, X_i$ and $Y_i$ as Alice does in Step 5.1 of Protocol 1. $\mathcal {R}$ then performs Eve’s actions in the protocol (which only act on $Y_i$ and $E'_{i-1}$, which is part of $E_{i-1}$). It is clear that the distribution on $X_i$ and $Y_i$ produced by $\mathcal {R}_i$ is the same as for $\mathcal {N}_i$. By the assumption that *D* and Eve cannot communicate, the marginal of the output of $\mathcal {N}_i$ on Eve’s side must be independent of the device’s system $R_{i-1}$. Hence, $\text {Tr}_{A_i R_i C_i} \circ \mathcal {N}_i = \mathcal {R}_i \circ \text {Tr}_{R_{i-1}}$.

To construct a min-tradeoff function, we note that we can split $\mathcal {N}_i = \gamma \mathcal {N}_i^{\text {test}} + (1-\gamma ) \mathcal {N}_i^{\text {data}}$, with $\mathcal {N}_i^{\text {test}}$ always picking $T_i = 1$ and $\mathcal {N}_i^{\text {data}}$ always picking $T_i = 0$. Then, we get from Lemma 4.7 and the condition $\textsf{Max}(g) = g(\delta _1)$ that the affine function *f* defined by

$$\begin{aligned} f(\delta _0) = g(\delta _1) + \frac{1}{\gamma }(g(\delta _0) - g(\delta _1)) \,, \qquad f(\delta _1) = f(\delta _\bot ) = g(\delta _1) \end{aligned}$$

is an affine min-tradeoff function for $\{\mathcal {N}_i\}$.

Viewing the event $\Omega$ as a subset of the range $\{0, 1\}^n$ of the random variable $C^n$ and comparing with the abort condition in Protocol 1, we see that $c^n \in \Omega$ implies $\textsf{freq}(c^n)(0) \le (1 - \omega _{\text {exp}}+ \delta ) \gamma$. Therefore, for $c^n \in \Omega$ and denoting $p = \textsf{freq}(c^n)$,

$$\begin{aligned} f(\textsf{freq}(c^n)) = p(0) f(\delta _0) + (1 - p(0)) f(\delta _1) = \frac{p(0)}{\gamma } g(\delta _0) + \left( 1- \frac{p(0)}{\gamma } \right) g(\delta _1) \ge h \,, \end{aligned}$$

where the last inequality holds because *g* is affine and the distribution $p'(0) = p(0)/\gamma , p'(1) = 1-p(0)/\gamma$ satisfies $p'(0) \le 1 - \omega _{\text {exp}}+ \delta$. The proposition now follows directly from Corollary 4.6 and the scaling of $c_1$ and $c_0$ is easily obtained from the expressions in Corollary 4.6.

To show that an honest strategy succeeds in the protocol with high probability, we define a random variable $F_i$ by $F_i = 1$ if $C_i = 0$, and $F_i = 0$ otherwise. If *D* and Eve execute the quantum strategy that wins the game *G* with probability $\omega _{\text {exp}}$ in each round, then $\mathop {\mathrm {\mathbb {E}}}\limits [F_i] = (1 - \omega _{\text {exp}}) \gamma$. Using the abort condition in the protocol, we then find

$$\begin{aligned} \textrm{Pr}\!\left[ \text {abort} \right]&= \textrm{Pr}\!\left[ \sum _{i=1}^n F_i> (1 - \omega _{\text {exp}}+ \delta )\cdot \gamma n \right] \\&= \textrm{Pr}\!\left[ \sum _{i=1}^n F_i > \left( 1 + \frac{\delta }{1-\omega _{\text {exp}}} \right) \cdot \mathop {\mathrm {\mathbb {E}}}\limits \Big [ \sum _{i=1}^n F_i \Big ] \right] \\&\le e^{-\frac{\delta ^2}{1 - \omega _{\text {exp}}+ \delta } \gamma n} \,, \end{aligned}$$

where in the last line we used a Chernoff bound. $\square$

**Fig. 1:**
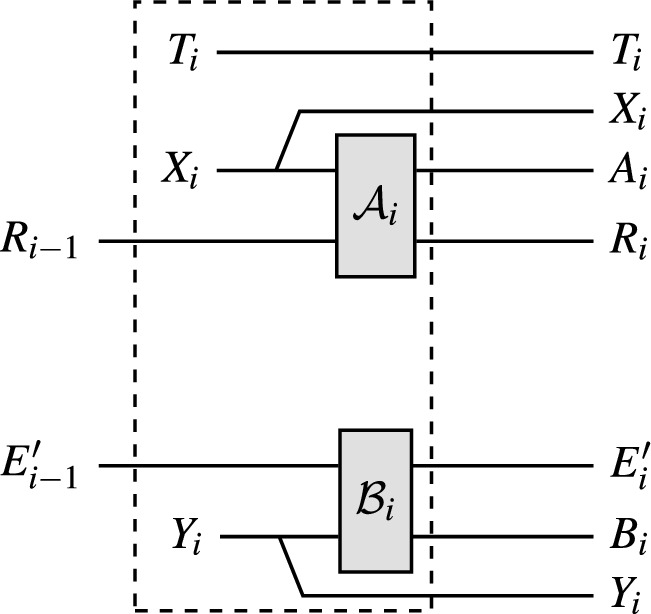
Circuit diagram of $\mathcal {N}: R_{i-1} E'_{i-1} \rightarrow A_iR_iT_iX_iY_iB_iE'_i$. For every round of the protocol, a circuit of this form is applied, where $\mathcal {A}$ and $\mathcal {B}$ are the (arbitrary) channels applied by Alice’s device and Eve, respectively. As in the protocol, $T_i$ is a bit equal to 1 with probability $\gamma$, and $X_i$ and $Y_i$ are generated according to *q* whenever $T_i=1$, and are fixed to $x^*,y^*$ otherwise. We did not include the register $C_i$ in the figure as it is a deterministic function of $T_iX_iY_iA_iB_i$

To make use of Lemma 5.1, we need to construct a function *g*(*p*) that satisfies the condition in Eq. (5.1). For this, we will use the equivalent condition Eq. (5.2). A general way of obtaining such a bound automatically is using the recent numerical method [22].^14^ Specifically, using the assumption that Alice’s lab is isolated, the maps $\mathcal {N}_i$ describing a single round of the protocol take the form described in Fig. 1.

**Fig. 2:**
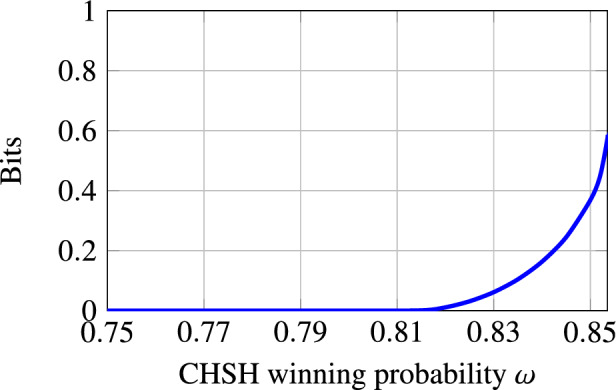
Lower bound on the conditional entropy $H(A_i|B_iX_iY_iT_iE_i')_{\rho _{|T_i=0}}$ for any state generated as in Fig. 1 and such that on test rounds the obtained winning probability for the CHSH game is $\omega$. This lower bound was obtained by using the method from [22]. For each input $y \in \mathcal {Y}$, the channel $\mathcal {B}_{y}$ is modelled as $\mathcal {B}_y(\omega ) = \sum _{b} \Pi _y^{(b)} \omega \Pi _y^{(b)}$, where $\{\Pi _y^{(b)}\}_{b \in \mathcal {B}}$ are orthogonal projectors summing to the identity, and similarly for the map $\mathcal {A}$. It is simple to see that this is without loss of generality

The method of [22] allows one to obtain lower bounds on the infimum of

$$\begin{aligned} H(A_i|B_iX_iY_iT_iE'_i\tilde{E}_{i-1})_{\mathcal {N}_i(\omega _{R_{i-1}E'_{i-1}{{\tilde{E}}}_{i-1}})} \end{aligned}$$

over all input states $\omega _{R_{i-1}E'_{i-1}{{\tilde{E}}}_{i-1}}$ and for any map $\mathcal {N}_i$ of the form depicted in Fig. 1. Importantly, for any $\mathcal {N}_i$ we may also restrict the infimum to states $\omega$ that are consistent with the observed statistics, i.e., $\mathcal {N}^{\text {test}}(\omega )_{C_i} = p$ for some distribution *p* on $C_i$, using the notation of Lemma 5.1. Using this numerical method for the CHSH game, we obtain the values shown in Fig. 2. From this, one can also construct an explicit affine min-tradeoff function *g*(*p*) in an automatic way using the same method as in [46]. As our focus is on illustrating the use of the generalised EAT, not the single-round bound, we do not carry out these steps in detail here.

Combining this single-round bound and Lemma 5.1, one obtains that for Protocol 1 instantiated with the CHSH game, $\omega _{\text {exp}}$ sufficiently close to the maximal winning probability of $\frac{1}{2} + \frac{1}{2\sqrt{2}}$, and $\gamma = \Theta (\frac{\log n}{n})$, one can extract $\Omega (n)$ bits of uniform randomness from $A_1 \dots A_n$ while using only $\text {polylog}(n)$ bits of randomness to run the protocol. In other words, Protocol 1 achieves exponential blind randomness expansion with the CHSH game.

### E91 quantum key distribution protocol

The E91 protocol is one of the simplest entanglement-based QKD protocols [45, 47]. This protocol was already treated using the original EAT in [1]. Here, we do not give a formal security definition and proof, only an informal comparison of how the original EAT and our generalised EAT can be applied to this problem; the remainder of the security proof is then exactly as in [1]. For a detailed treatment of the application of our generalised EAT to QKD, see [7]. To facilitate the comparison with [1], in this section we label systems the same as in [1] even though this differs from the system labels used earlier in this paper. The protocol we are considering is described explicitly in Protocol 2. It is the same as in [1] except for minor modifications to simplify the notation.
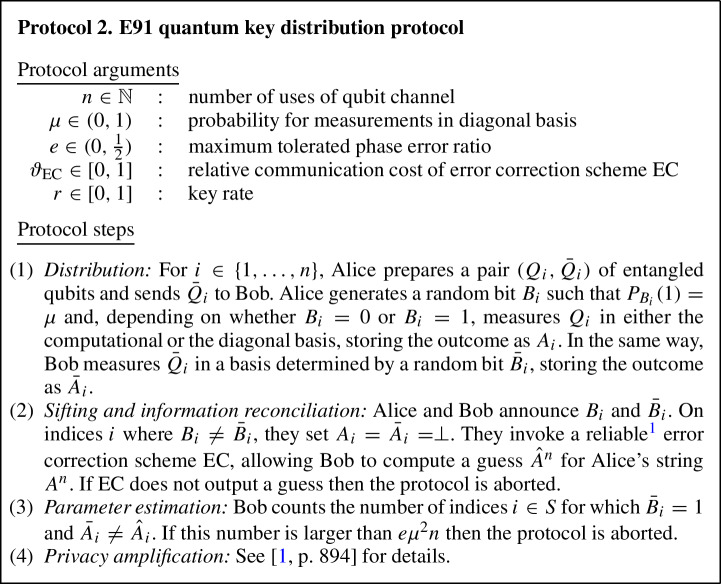


We consider the systems $B_i, {\bar{B}}_i, A_i, {\bar{A}}_i, Q_i, \bar{Q}_i$ as in Protocol 2 and additionally define the system $X_i$ storing the statistical information used in the parameter estimation step:

$$\begin{aligned} X_i = {\left\{ \begin{array}{ll} A_i \oplus {\bar{A}}_i &  \text {if }B_i = {\bar{B}}_i = 1,\\ \bot &  \text {otherwise.} \end{array}\right. } \end{aligned}$$

Denoting by *E* the side information gathered by Eve during the distribution step, we can follow the same steps as for [1, Equation (57)] to show that the security of Protocol 2 follows from a lower bound on

5.3 $$\begin{aligned} H_\text {min}^\varepsilon (A^n | B^n {\bar{B}}^n E)_{\rho _{|\Omega }} \,. \end{aligned}$$

Here, $\rho _{|\Omega }$ is the state at the end of the protocol conditioned on acceptance.

We first sketch how the original EAT (whose setup was described in Sect. 1) is applied to this problem in [1]. One cannot bound $H_\text {min}^\varepsilon (A^n | B^n {\bar{B}}^n E)_{\rho _{|\Omega }}$ directly using the EAT because a condition similar to Eq. (4.2) has to be satisfied. Therefore, one modifies the systems ${\bar{A}}_i$ from Protocol 2 by setting ${\bar{A}}_i = \bot$ if $B_i = {\bar{B}}_i = 0$ and then applies the EAT to find a lower bound on

5.4 $$\begin{aligned} H_\text {min}^\varepsilon (A^n {\bar{A}}^n | B^n {\bar{B}}^n E)_{\rho _{|\Omega }} \,. \end{aligned}$$

For this, a round of Protocol 2 is viewed as a map $\mathcal {M}_i: Q_i^n {\bar{Q}}_i^n \rightarrow Q_{i+1}^n {\bar{Q}}_{i+1}^n A_i {\bar{A}}_i B_i \bar{B}_i X_i$, which chooses $B_i {\bar{B}}_i$ as in Protocol 2, applies Alice and Bob’s (trusted) measurements on systems $Q_i \bar{Q}_i$ to generate $A_i {\bar{A}}_i$, and generates $X_i$ as described before. To apply the EAT, $R_{i-1} {:}{=}Q_i^n {\bar{Q}}_i^n$ takes the role of the “hidden sytem”, and $A_i {\bar{A}}_i$ and $B_i {\bar{B}}_i$ are the output and side information of the *i*-th round, respectively. It is easy to see that with this choice of systems, the Markov condition of the EAT is satisfied, so, using a min-tradeoff function derived from an entropic uncertainty relation [48], one can find a lower bound on Eq. (5.4).

However, adding the system ${\bar{A}}_i$ in this manner has the following disadvantage: to relate the lower bound on $H_\text {min}^\varepsilon (A^n {\bar{A}}^n | B^n {\bar{B}}^n E)_{\rho _{|\Omega }}$ to the desired lower bound on $H_\text {min}^\varepsilon (A^n | B^n {\bar{B}}^n E)_{\rho _{|\Omega }}$ one needs to use a chain rule for min-entropies, incurring a penalty term of the form $H_{\text {max}}^\varepsilon ({\bar{A}}^n | A^n B^n {\bar{B}}^n E)_{\rho _{|\Omega }}$. This penalty term is relatively easy to bound for the case of the E91 protocol, but can cause problems in general.^15^

We now turn our attention to proving Eq. (5.3) using our generalised EAT. For this, we first observe that

$$\begin{aligned} H_\text {min}^\varepsilon (A^n | B^n {\bar{B}}^n E)_{\rho _{|\Omega }} \ge H_\text {min}^\varepsilon (A^n | B^n {\bar{B}}^n X^n E)_{\rho _{|\Omega }} \,, \end{aligned}$$

so it suffices to find a lower bound on the r.h.s. This step is similar to adding the ${\bar{A}}_i$ systems in Eq. (5.4) in that its purpose is to satisfy Eq. (4.2). However, it has the advantage that here, $X^n$ can be added to the *conditioning* system and therefore lowers the entropy, not raises it like going from Eqs. (5.3) to (5.4). The same step is not possible in the original EAT due to the restrictive Markov condition.

Using the same system names as before, we define $E_i {:}{=}Q_{i+1}^n {\bar{Q}}_{i+1}^n B^i {\bar{B}}^i X^i E$.^16^ Then, analogously to the original EAT, we can describe a single round of Protocol 2 by a map $\mathcal {M}_i: E_{i-1} \rightarrow A_i E_{i} X_i$. (Compared to the map $\mathcal {M}_i$ we described above for the original EAT, we have traced out ${\bar{A}}_i$, added a copy of $X_i$, and added identity maps on the other additional systems in $E_{i-1}$.) Denoting by $\rho ^0_{Q^n {\bar{Q}}^n E}$ the joint state of Alice and Bob’s systems $Q^n {\bar{Q}}^n$ before measurement and the information *E* that Eve gathered during the distribution step, the state at the end of the protocol is $\rho = \mathcal {M}_n \circ \dots \circ \mathcal {M}_1(\rho ^0)$. To apply Corollary 4.6 to find a lower bound on

$$\begin{aligned} H_\text {min}^\varepsilon (A^n | E_n)_{\mathcal {M}_n \circ \dots \circ \mathcal {M}_1(\rho ^0)_{|\Omega }} \,, \end{aligned}$$

we first observe that the condition in Eq. (4.2) is satisfied because the system $X^n$ is part of $E_n$, and the non-signalling condition is trivially satisfied because there is no $R_i$-system. A min-tradeoff function can be constructed in exactly the same way as in [1, Claim 5.2] by noting that all systems in $E_i$ on which $\mathcal {M}_i$ does not act can be viewed as part of the purifying system.

This comparison highlights the advantage of the more general model of side information in our generalised EAT: for the original EAT, one has to first bound $H_\text {min}^\varepsilon (A^n {\bar{A}}^n | B^n {\bar{B}}^n E)$ (rather than $H_\text {min}^\varepsilon (A^n | B^n {\bar{B}}^n E)$) in order to be able to satisfy the Markov condition, and then perform a separate step to remove the ${\bar{A}}^n$ system. In our case, the non-signalling condition, the analogue of the Markov condition, is trivially satisfied because we need no $R_i$-system. This is because we can add the quantum systems $Q^n {\bar{Q}}^n$ to the side information register $E_0$ at the start and then, since we allow side information to be updated and Alice and Bob act on $Q_i {\bar{Q}}_i$ using trusted measurement devices, we can remove the systems $Q_i {\bar{Q}}_i$ one by one during the rounds of the protocol.

## Data Availability

No experimental data has been generated as part of this project. The introduction of this work has been published as an extended abstract in the proceedings of FOCS 2022 [49].

## Dual Statement for Smooth Max-Entropy

In the main text we have focused on deriving a lower bound on the smooth min-entropy. Here, we show that this also implies an upper bound on the smooth max-entropy by applying a simple duality relation between min- and max-entropy. A similar upper bound was also derived in [1]. However, that bound is subject to a Markov condition and cannot be derived by a simple duality argument since the “dual version” of the Markov condition is unwieldy. We show that the bound from [1] follows as a special case of our more general bound even without any Markov conditions or other non-signalling constraints. For simplicity, we restrict ourselves to an asymptotic statement without “testing”, i.e. we derive an $H_{\text {max}}^\varepsilon$-version of Theorem 4.1. By applying the same duality relation to the more involved statement in Theorem 4.3, one can also obtain an $H_{\text {max}}^\varepsilon$-bound with explicit constants and testing.

Recall that for $\rho _{AB} \in \text {S}(AB)$ and $\varepsilon \in [0,1]$, the $\varepsilon$-smoothed max-entropy of *A* conditioned on *B* is defined as

$$\begin{aligned} H_{\max }^\varepsilon (A|B)_{\rho } = \log \inf _{{\tilde{\rho }}_{AB} \in \mathcal {B}_{\varepsilon }(\rho _{AB})} \sup _{\sigma _{B} \in \text {S}(B)} \left\Vert \tilde{\rho }_{AB}^{\frac{1}{2}} \sigma _B^{\frac{1}{2}} \right\Vert _{1}^2 \, , \end{aligned}$$

where $\left\Vert \cdot \right\Vert _{1}$ denotes the trace norm and $\mathcal {B}_{\varepsilon }(\rho _{AB})$ is the $\varepsilon$-ball around $\rho _{AB}$ in terms of the purified distance [11]. The smooth min- and max-entropy satisfy the following duality relation [11, Proposition 6.2]: for a pure quantum state $\psi _{ABC}$,

$$\begin{aligned} H_\text {min}^\varepsilon (A|B)_{\psi } = - H_{\text {max}}^\varepsilon (A|C)_{\psi } \,. \end{aligned}$$

For the setting of Theorem 4.1, let $V_i: R_{i-1} E_{i-1} \rightarrow A_i R_i E_i F_i$ be the Stinespring dilation of the map $\mathcal {M}_i$, and let $|\rho ^0\rangle _{R_0 E_0 F_0}$ be a purification of the input state $\rho ^0_{R_0 E_0}$. Then, $V_n \cdots V_1 |\rho ^0\rangle$ is a purification of $\mathcal {M}_n \circ \dots \circ \mathcal {M}_1(\rho ^0)$, so by the duality of the smooth min- and max-entropy,

$$\begin{aligned} H_\text {min}^\varepsilon (A^n | E_n )_{\mathcal {M}_n \circ \dots \circ \mathcal {M}_1(\rho ^0)} = - H_{\text {max}}^\varepsilon (A^n | F^n R_n)_{V_n \cdots V_1 |\rho ^0\rangle } \,. \end{aligned}$$

Furthermore, by concavity of the conditional entropy the infimum in Theorem 4.1 can be restricted to pure states $|\omega \rangle _{R_{i-1} E_{i-1} {{\tilde{E}}}_{i-1}}$, so $V_i |\omega \rangle$ is a purification of $\mathcal {M}_i(\omega )$. Then, by the duality relation for von Neumann entropies,

$$\begin{aligned} H(A_i|E_i {{\tilde{E}}}_{i-1} )_{\mathcal {M}_i(\omega )} = - H(A_i|R_i F_i)_{V_i |\omega \rangle } \,. \end{aligned}$$

Therefore, we obtain the following dual statement to Theorem 4.1:

A.1 $$\begin{aligned} H_{\text {max}}^\varepsilon (A^n | F^n R_n)_{V_n \cdots V_1 |\rho ^0\rangle } \le \sum _{i = 1}^n \max _{|\omega \rangle } H(A_i|R_i F_i)_{V_i |\omega \rangle } + O(\sqrt{n}) \,, \end{aligned}$$

where the maximisation is over pure states on $R_{i-1} E_{i-1} {{\tilde{E}}}_{i-1}$. This holds for any sequence of isometries $V_i$ for which the maps $\mathcal {M}_{V_i}: R_{i-1} E_{i-1} \rightarrow A_i R_i E_i$ given by $\mathcal {M}_{V_i}(\rho ) = \text{ Tr}_{F_i}\!\left[ V_i \rho V_i^\dagger \right]$ satisfy the non-signalling condition of Theorem 4.1: for each *i*, there must exist a map $\mathcal {R}_i \in \text {CPTP}(E_{i-1}, E_i )$ such that $\text {Tr}_{A_i R_i} \circ \mathcal {M}_{V_i} = \mathcal {R}_i \circ \text {Tr}_{R_{i-1}}$.

To gain some intuition for the above statement, consider a setting where an information source generates systems $A_1, \dots , A_n$ and $F_1, \dots , F_n$ by applying isometries $V_i: S_{i-1} \rightarrow A_i F_i S_i$ to some pure intial state $|\rho ^0\rangle _{S_0}$. We might be interested in compressing the information in $A^n$ in such a way that given $F^n$, one can reconstruct $A^n$ except with some small failure probability $\varepsilon$. Then, the amount of storage needed for the compressed information is given by $H_{\text {max}}^\varepsilon (A^n|F^n)$. To apply Eq. (A.1), for $i < n$ we split the systems $S_i$ into $R_i E_i$ in such a way that the channel $\mathcal {M}_{V_i}$ defined above satisfies the non-signalling condition, and set $E_n = S_n$ (so that $R_n$ is trivial). Then Eq. (A.1) gives an upper bound on $H_{\text {max}}^\varepsilon (A^n|F^n)$. Note that this bound depends on how we split the systems $S_i = R_i E_i$: the non-signalling condition can always be trivially satisfied by choosing $R_i$ to be trivial, but Eq. (A.1) tells us that if we can describe the source in such a way that $E_i$ is relatively small and $R_i$ is relatively large while still satisfying the non-signalling condition, we obtain a tighter bound on the amount of required storage.

From Eq. (A.1) we can also recover the max-entropy version of the original EAT, but without requiring a Markov condition. To facilitate the comparison with [1], we first re-state their theorem with their choice of system labels, but add a bar to every system label to avoid confusion with our notation from before. The max-entropy statement in [1] considers a sequence of channels ${\bar{\mathcal {M}}}_i: {\bar{R}}_{i-1} \rightarrow {\bar{A}}_i {\bar{B}}_i {\bar{R}}_i$ and asserts that under a Markov condition, for any initial state $\rho _{{\bar{R}}_0 {\bar{E}}}$ with a purifying system ${\bar{E}} \equiv \bar{R}_0$:

A.2 $$\begin{aligned} H_{\text {max}}^\varepsilon ({\bar{A}}^n | {\bar{B}}^n {\bar{E}})_{{\bar{\mathcal {M}}}_n \circ \cdots \circ {\bar{\mathcal {M}}}_1(\rho _{{\bar{R}}_0 {\bar{E}}})} \le \sum _{i=1}^n \max _{\omega \in \text {S}({\bar{R}}_{i-1} {\bar{R}})} H({\bar{A}}_i | {\bar{B}}_i {\bar{R}})_{{\bar{\mathcal {M}}}_i(\omega )} + O(\sqrt{n}) \,, \end{aligned}$$

where ${\bar{R}} \equiv {\bar{R}}_{i-1}$. We want to recover this statement from Eq. (A.1) *without any Markov condition*. For this, we consider the Stinepring dilations $\bar{V}_i: {\bar{R}}_{i-1} \rightarrow {\bar{R}}_i {\bar{A}}_i {\bar{B}}_i {\bar{F}}_i$ of $\bar{\mathcal {M}}_i$. We make the following choice of systems:

$$\begin{aligned} R_i = {\bar{B}}^i {\bar{E}} \,, \quad A_i = {\bar{A}}_i \,, \quad E_i = \bar{R}_i {\bar{F}}_i \,, \end{aligned}$$

and choose $F_i$ to be trivial. By tensoring with the identity, we can then extend ${\bar{V}}_i$ to an isometry $V_i: R_{i-1} E_{i-1} \rightarrow A_i R_i E_i$. Then, the maps $\mathcal {M}_{V_i}$ satisfy the non-signalling condition since $V_i$ acts as identity on $R_{i-1}$. Therefore, remembering that $R_n = {\bar{B}}^n {\bar{E}}$ and $F^n$ is trivial, we see that Eq. (A.1) implies Eq. (A.2). Note that our derivation did not require any conditions on the channels ${\bar{\mathcal {M}}}_i$ we started with, i.e. we have shown Eq. (A.2) holds for any sequence of channels $\bar{\mathcal {M}}_i$, not just channels satisfying a Markov or non-signalling condition.

## Uhlmann Property for the Rényi Divergence

We establish that for the max-divergence (where $\alpha \rightarrow \infty$), Uhlmann’s theorem holds.

### Proposition B.1

Let $\sigma _{A} \in \text {S}(A)$ and $\rho _{AR} \in \text {S}(A R)$. Then we have

B.1 $$\begin{aligned} D_{\max }(\rho _{A} \Vert \sigma _{A}) = \inf _{\hat{\sigma }_{AR} \, : \, \hat{\sigma }_{A} = \sigma _{A}} D_{\max }(\rho _{AR} \Vert \hat{\sigma }_{AR}) \ . \end{aligned}$$

In addition, if $\rho _{AR}, \rho _{A} \otimes \text {id}_{R}$ and $\sigma _{A} \otimes \text {id}_{R}$ all commute, then for any $\alpha \in [\frac{1}{2}, \infty )$, we have

B.2 $$\begin{aligned} D_{\alpha }(\rho _{A} \Vert \sigma _{A}) = \inf _{\hat{\sigma }_{AR} \, : \, \hat{\sigma }_{A} = \sigma _{A}} D_{\alpha }(\rho _{AR} \Vert \hat{\sigma }_{AR}) \, . \end{aligned}$$

### Proof

We start with Eq. (B.1). The inequality $\le$ is a direct consequence of the data-processing inequality for $D_{\max }$. For the inequality $\ge$, we use semidefinite programming duality, see e.g., [50]. Observe that we can write $2^{D_{\max }( \rho _{A} \Vert \sigma _{A})}$ as the following semidefinite program

$$\begin{aligned} \min _{\tau _{A} \in {{\,\textrm{Pos}\,}}(A), \lambda \in \mathbb {R}} \{ \text{ Tr }\!\left[ \tau _{A} \right] \; \text { subject to } \; \rho _{A} \le \tau _{A} \, \text { and } \, \tau _{A} = \lambda \sigma _{A} \} \, . \end{aligned}$$

Using semidefinite programming duality, this is also equal to

B.3 $$\begin{aligned} \max _{X_{A} \in {{\,\textrm{Pos}\,}}(A), Y_{A} \in \text {Herm}(A) } \{ \text{ Tr }\!\left[ X_{A} \rho _{A} \right] \; \text { subject to } \; \text {id}_{A} + Y_{A} = X_{A} \, \text { and } \, \text{ Tr }\!\left[ Y_{A} \sigma _{A} \right] = 0 \} \, . \end{aligned}$$

We can also write a semidefinite program for $\inf _{\hat{\sigma }_{AR} \,: \, \hat{\sigma }_{A} = \sigma _A} 2^{ D_{\max }(\rho _{AR} \Vert \hat{\sigma }_{AR}) }$. We introduce the variable $\theta _{AR} = \lambda \hat{\sigma }_{AR}$ and get

$$\begin{aligned} \min _{\theta \in {{\,\textrm{Pos}\,}}(A \otimes R), \lambda \in \mathbb {R}} \{ \text{ Tr }\!\left[ \theta _{AR} \right] \; \text { subject to } \; \rho _{AR} \le \theta _{AR} \, \text { and } \, \theta _{A} = \lambda \sigma _{A} \} \, . \end{aligned}$$

Again, by semidefinite programming duality, we get that it is equal to

B.4 $$\begin{aligned}&\max _{X_{AR} \in {{\,\textrm{Pos}\,}}(A \otimes R), Y_{A} \in \text {Herm}(A)} \{ \text{ Tr }\!\left[ X_{AR} \rho _{AR} \right] \; \text { subject to } \; (\text {id}_{A} + Y_{A}) \otimes \text {id}_{R} \nonumber \\&\quad = X_{AR} \, \text { and } \, \text{ Tr }\!\left[ Y_{A} \sigma _{A} \right] = 0 \}\, . \end{aligned}$$

Eliminating the variable $X_{AR}$, we can write this last program as

$$\begin{aligned} \max _{Y_{A} \in \text {Herm}(A)} \{ \text{ Tr }\!\left[ (\text {id}_{A} + Y_{A}) \rho _{A} \right] \; \text { subject to } \; \text {id}_{A} + Y_{A} \in {{\,\textrm{Pos}\,}}(A) \, \text { and } \, \text{ Tr }\!\left[ Y_{A} \sigma _{A} \right] = 0 \}\, , \end{aligned}$$

which is the same as Eq. (B.3). This proves Eq. (B.1). Equation (B.2) follows immediately by choosing $\hat{\sigma }_{AR} = \sigma _{A}\rho _{A}^{-1}\rho _{AR}$ and using the commutation conditions. $\square$

However, for $\alpha \ge 1$ and arbitrary $\sigma _{A} \in \text {S}(A)$, $\rho _{AE} \in \text {S}(A E)$, the Uhlmann property given by Eq. (B.2) does not hold. A concrete example is $\rho _{AR} = |\psi \rangle \!\langle \psi |_{AR}$ with

$$\begin{aligned} |\psi \rangle _{AR} = \sqrt{\frac{1}{4}} |00\rangle _{AR} + \sqrt{\frac{3}{4}} |11\rangle _{AR} \end{aligned}$$

and $\sigma _{A} = \frac{1}{3} |+\rangle \!\langle +| + \frac{2}{3} |-\rangle \!\langle -|$. In this case, $D_2(\rho _A \Vert \sigma _A) < 0.476$ whereas

$$\begin{aligned} \inf _{\hat{\sigma }_{AR} \, : \, \hat{\sigma }_{A} = \sigma _{A}} D_2(\rho _{AR} \Vert \hat{\sigma }_{AR} ) \ge \inf _{\hat{\sigma }_{AR} \, : \, \hat{\sigma }_{A} = \sigma _{A}} D(\rho _{AR} \Vert \hat{\sigma }_{AR}) > 0.48 \, . \end{aligned}$$

This computation was performed by numerically solving the semidefinite programs via CVXQUAD [51]. Putting everything together shows that Eq. (B.2) does not hold for $\alpha \in \{1,2\}$:

$$\begin{aligned} D(\rho _{A} \Vert \sigma _{A}) \le D_2(\rho _A \Vert \sigma _A) < \inf _{\hat{\sigma }_{AR} \, : \, \hat{\sigma }_{A} = \sigma _{A}} D(\rho _{AR} \Vert \hat{\sigma }_{AR}) \le \inf _{\hat{\sigma }_{AR} \, : \, \hat{\sigma }_{A} = \sigma _{A}} D_{2}(\rho _{AR} \Vert \hat{\sigma }_{AR}) \, . \end{aligned}$$

## Footnotes

1 Since $\rho$ is a product of identical states, all of the terms $H(A_i | E_i)_\rho$ are equal, i.e., $\sum _{i=1}^n H(A_i | E_i)_\rho = n H(A_i | E_i)_\rho$ for any *i*. We write the sum here explicitly to highlight the analogy with the EAT presented below.

2 The EAT from [1] also makes an analogous statement about an upper bound on the max-entropy $H_{\text {max}}$. We derive a generalisation of that statement in Appendix A but only focus on $H_\text {min}$ in the introduction and main text since that is the case that is typically relevant for applications.

3 In fact, the EAT is more general in that it allows taking into account observed statistics to restrict the minimization over $\omega _{A_i B_i E}$, but we restrict ourselves to the simpler case without statistics in this introduction.

4 As usual, the channels $\mathcal {M}_i$ act as identity on any additional systems that may be part of the input state, i.e. $\mathcal {M}_i(\omega _{R_{i-1}E_{i-1}{{\tilde{E}}}_{i-1}}) = (\mathcal {M}_i \otimes \text {id}_{{{\tilde{E}}}_{i-1}})(\omega _{R_{i-1}E_{i-1}{{\tilde{E}}}_{i-1}})$ is a state on $A_i R_i E_i {{\tilde{E}}}_{i-1}$. In particular, the register ${{\tilde{E}}}_{i-1}$ containing a purification of the input is also part of the output state.

5 Strictly speaking, the EAT as stated in [1] only requires that this Markov property holds for any input state $\omega ^{i-1}$ in the image of the previous maps $\mathcal {M}_{i-1} \circ \dots \circ \mathcal {M}_1$. The same is true for the non-signalling condition, i.e., one can check that our proof of the generalised EAT still works if the map $\mathcal {R}_i$ only satisfies Eq. (1.2) on states in the image of $\mathcal {M}_{i-1} \circ \dots \circ \mathcal {M}_1$. To simplify the presentation, we use the stronger condition Eq. (1.2) throughout this paper.

6 We note that the definition of Rényi entropies can be extended to $\alpha < 1$, but we will only need the case $\alpha > 1$.

7 In fact, in order for this single-round quantity to be positive one has to restrict the infimum to input states that allow the non-local game to be won with a certain probability. This requires using the generalised EAT with testing (Sect. 4.2), not Theorem 1.1. We refer to Sect. 5.1 for details.

8 In an EAT-like theorem, the entropy contribution from a particular round *i* has to be calculated conditioned on the side information revealed in that round because we want to analyse the process round-by-round, not globally. If a future round revealed additional side information, then the total entropy contributed by round *i* would decrease, but there is no way of accounting for that in an EAT-like theorem that simply sums up single-round contributions. As an extreme case, the last round of the process could reveal all prior outputs as side information, so that the total amount of conditional entropy produced by the process is 0, but single-round entropy contributions could be positive. This demonstrates the need for some condition that enforces that future side information does not reveal information about past outputs. We note that this does not mean that there is no way of proving an entropy lower bound in more general settings: for example, [32] do show a bound on the entropy produced by parallel repeated non-local games, but this requires a global analysis.

9 “Stabilised” refers to the fact that the supremum in Eq. (2.1) maximises over states in $\text {S}(A {{\tilde{A}}})$, not just $\text {S}(A)$, i.e. the maximisation includes a purifying system ${{\tilde{A}}}$. One can also consider non-stabilised channel divergences, where the supremum is only over states in $\text {S}(A)$. However, in this paper we only use the stabilised channel divergence.

10 It is well-known [3, Lemma 8] that $\lim _{\alpha \searrow 1} D_\alpha \! \left( \mathcal {E} \, \big \Vert \, \mathcal {F} \right) = D\big (\mathcal {E}\Vert \mathcal {F}\big )$, but it is unclear whether the same holds for the regularised quantity.

11 In case ${\hat{\rho }}_{A^n}$ does not have full support, we only take the inverse on the support of ${\hat{\rho }}_{A^n}$.

12 The map $\mathcal {M}$ in the theorem statement is also implicitly tensored with an identity map on *A*, but for the definition of ${{\tilde{M}}}$ we make this explicit to avoid confusion when applying Theorem 3.1.

13 A function *f* on the convex set $\mathbb {P}(\mathcal {C})$ is called *affine* if it is linear under convex combinations, i.e., for $\lambda \in [0,1]$ and $p_1, p_2 \in \mathbb {P}(\mathcal {C})$, $\lambda f(p_1) + (1 - \lambda ) f(p_2) = f(\lambda p_1 + (1 - \lambda ) p_2)$. Such functions are also sometimes called *convex-linear*.

14 The main result of [15] (Theorem 14) does not appear to be sufficient for this. The reason is that the statement made in [15] essentially concerns the randomness produced on average over the question distribution *q* of the game *G*. However, choosing a question at random consumes randomness, so to achieve exponential randomness expansion, in Protocol 1 we fix the inputs $x^*, y^*$ used for generation rounds. To the best of our knowledge, the results of [15] do not give a bound on the randomness produced in the non-local game for any *fixed* inputs $x^*, y^*$. If one could prove an analogous statement to [15, Theorem 14] that also certifies randomness on fixed inputs for a large class of games, our Lemma 5.1 would then imply exponential blind randomness expansion for any such game. Alternatively, one can also assume that public (non-blind) randomness is a free resource and use this to choose the inputs for the non-local game. Then, no special inputs $x^*, y^*$ are needed in Protocol 1 to “save randomness” and the result of [15] combined with our generalised EAT implies that such a conversion from public to blind randomness is possible for any complete-support game.

15 An error correction scheme is reliable if, except with negligible probability, either Bob’s guess of Alice’s string is correct or the protocol aborts.

16 In Protocol 2, instead of Alice distributing the systems $Q_i {\bar{Q}}_i$ and Eve gathering side information *E* by intercepting ${\bar{Q}}_i$, we can equivalently imagine that Eve first prepares a state $\rho ^0_{Q^n {\bar{Q}}^n E}$ and distributes $Q_i {\bar{Q}}_i$ to Alice and Bob in each round. Then, the choice of $E_i$ intuitively captures the side information available to Eve from the first *i* rounds: Eve still possesses the systems $Q_{i+1}^n \bar{Q}_{i+1}^n$ to be distributed in future rounds, has gathered classical information $B^i {\bar{B}}^i X^i$, and keeps the static side information *E* from preparing the initial state.
